# A five amino acids deletion in NKCC2 of C57BL/6 mice affects analysis of NKCC2 phosphorylation but does not impact kidney function

**DOI:** 10.1111/apha.13705

**Published:** 2021-06-26

**Authors:** Sandra Moser, Yuya Sugano, Agnieszka Wengi, Viktoria Fisi, Lena Lindtoft Rosenbaek, Marta Mariniello, Dominique Loffing‐Cueni, James A. McCormick, Robert A. Fenton, Johannes Loffing

**Affiliations:** ^1^ Institute of Anatomy University of Zurich Zurich Switzerland; ^2^ Department of Biomedicine Aarhus University Aarhus Denmark; ^3^ Insitute of Physiology University of Zurich Zurich Switzerland; ^4^ Division of Nephrology and Hypertension Oregon Health & Science University Portland OR USA; ^5^ Swiss National Centre for Competence in Research “Kidney control of homeostasis” Zurich Switzerland

**Keywords:** ion homeostasis, kidney, NKCC2, phosphorylation, strain differences

## Abstract

**Aim:**

The phosphorylation level of the furosemide‐sensitive Na^+^‐K^+^‐2Cl^−^ cotransporter (NKCC2) in the thick ascending limb (TAL) is used as a surrogate marker for NKCC2 activation and TAL function. However, in mice, analyses of NKCC2 phosphorylation with antibodies against phosphorylated threonines 96 and 101 (anti‐pT96/pT101) give inconsistent results. We aimed (a) to elucidate these inconsistencies and (b) to develop a phosphoform‐specific antibody that ensures reliable detection of NKCC2 phosphorylation in mice.

**Methods:**

Genetic information, molecular biology, biochemical techniques and mouse phenotyping was used to study NKCC2 and kidney function in two commonly used mouse strains (ie 129Sv and in C57BL/6 mice). Moreover, a new phosphoform‐specific mouse NKCC2 antibody was developed and characterized.

**Results:**

Amino acids sequence alignment revealed that C57BL/6 mice have a strain‐specific five amino acids deletion (ΔF97‐T101) in NKCC2 that diminishes the detection of NKCC2 phosphorylation with previously developed pT96/pT101 NKCC2 antibodies. Instead, the antibodies cross‐react with the phosphorylated thiazide‐sensitive NaCl cotransporter (NCC), which can obscure interpretation of results. Interestingly, the deletion in NKCC2 does not impact on kidney function and/or expression of renal ion transport proteins as indicated by the analysis of the F2 generation of crossbred 129Sv and C57BL/6 mice. A newly developed pT96 NKCC2 antibody detects pNKCC2 in both mouse strains and shows no cross‐reactivity with phosphorylated NCC.

**Conclusion:**

Our work reveals a hitherto unappreciated, but essential, strain difference in the amino acids sequence of mouse NKCC2 that needs to be considered when analysing NKCC2 phosphorylation in mice. The new pNKCC2 antibody circumvents this technical caveat.

## 1 INTRODUCTION

The thick ascending limb (TAL) of the mammalian kidney is crucial for the control of extracellular fluid volume, urinary concentration, calcium (Ca^2+^) and magnesium (Mg^2+^) homeostasis, as well as pH balance.^1^ The major Na^+^ transport pathway in the TAL is the Na^+^‐K^+^‐2Cl^−^ cotransporter (NKCC2), which is specifically inhibited by loop‐diuretics.^1^ The cotransport of Na^+^, K^+^, and 2Cl^−^ ions via NKCC2 is electroneutral but depends on electrogenic K^+^ secretion via the apical renal outer medulla K^+^ channel ROMK.^2^ The interplay of NKCC2 with ROMK creates a lumen‐positive electrochemical gradient, that enhances Na^+^ reabsorption and drives the paracellular transport of Ca^2+^ and Mg^2+^.^3^ The significance of NKCC2 for TAL and kidney function is exemplified by Bartter type I syndrome, a genetic disorder caused by loss‐of‐function mutations in NKCC2, manifesting with severe renal salt and fluid wasting, hypokalemic metabolic alkalosis and hypercalciuria.^3^

NKCC2 is member of the solute carrier family 12 (*Slc12*) of membrane transport proteins that also includes the ubiquitously expressed NKCC1 and the distal convoluted tubule (DCT) specific Na^+^‐Cl^−^ cotransporter (NCC).^2^ The activity of NKCC2 positively correlates with phosphorylation of the cotransporter at several serine and threonine residues on the cytoplasmic N‐terminal tail.^4^ The amino acids sequences surrounding these phosphorylation sites are highly conserved and show a high degree of similarity among NKCC2, NKCC1 and NCC.^2^ The phosphorylation of NKCC2 is stimulated by several hormonal and non‐hormonal pathways. Hormones such as arginine vasopressin (AVP) and angiotensin II (ANGII) stimulate NKCC2 via increased cAMP production and activation of protein kinase A (PKA).^5^ PKA is thought to directly phosphorylate NKCC2 at Ser126, which enhances the exocytosis of NKCC2 containing vesicles and hence the abundance of the transporter in the apical plasma membrane.^6^ Likewise, changes in intracellular chloride concentrations regulate NKCC2 phosphorylation. A lowered intracellular Cl^−^ concentration stimulates the with‐no‐lysine(K) kinases WNK1 and WNK4 to phosphorylate and activate the downstream kinases Ste20‐related proline alanine rich kinase (SPAK) or oxidative stress responsive kinase 1 (OXSR1).^7^, ^8^, ^9^ After binding to a “RFxV” motif in NKCC2, these kinases then phosphorylate NKCC2 at two threonine residues (T96 and T101).^2^ Dephosphorylation of these residues is mediated by the phosphatase calcineurin, emphasizing that NKCC2 is a target for several signalling pathways.^10^

To study NKCC2 function, several groups including ours developed phosphoform‐specific antibodies against Thr96 and/or Thr101.^11^, ^12^, ^13^, ^14^, ^15^, ^16^, ^17^ The antibodies were used to assess NKCC2 phosphorylation in heterologous expression systems^7^, ^11^, ^17^, ^18^, ^19^, ^20^ and animal models including rats^10^, ^21^, ^22^, ^23^, ^24^, ^25^ and mice.^14^, ^15^, ^16^, ^17^, ^19^, ^26^, ^27^ However, when using our pT96/101 NKCC2 antibody, we obtained inconsistent results in our different mouse models. While we detected strong pNKCC2 signals in kidneys from mice in a 129Sv genetic background, we did not get reliable pNKCC2 signals in kidneys from C57BL/6 mice but observed a cross‐reactivity of the pNKCC2 antibodies with pNCC. Other laboratories, including the ones from J. A. McCormick and A. A. McDonough (personal communication) made similar observations.^18^, ^28^, ^29^ Furthermore, striking strain differences have been described in mice for renal function^30^ including significantly lower urinary Ca^2+^ excretion in C57BL/6 compared to 129Sv mice.^31^ Therefore, we hypothesized that C57BL/6 mice express a genetic variant of NKCC2 that impacts NKCC2 phosphorylation and possibly kidney function that explain the observed strain differences. The following study was designed to systematically test this hypothesis.

## 2 RESULTS

### 2.1 C57BL/6 mice have a five amino acids deletion in NKCC2 that interferes with the detection of phosphorylated NKCC2

**FIGURE 1:**
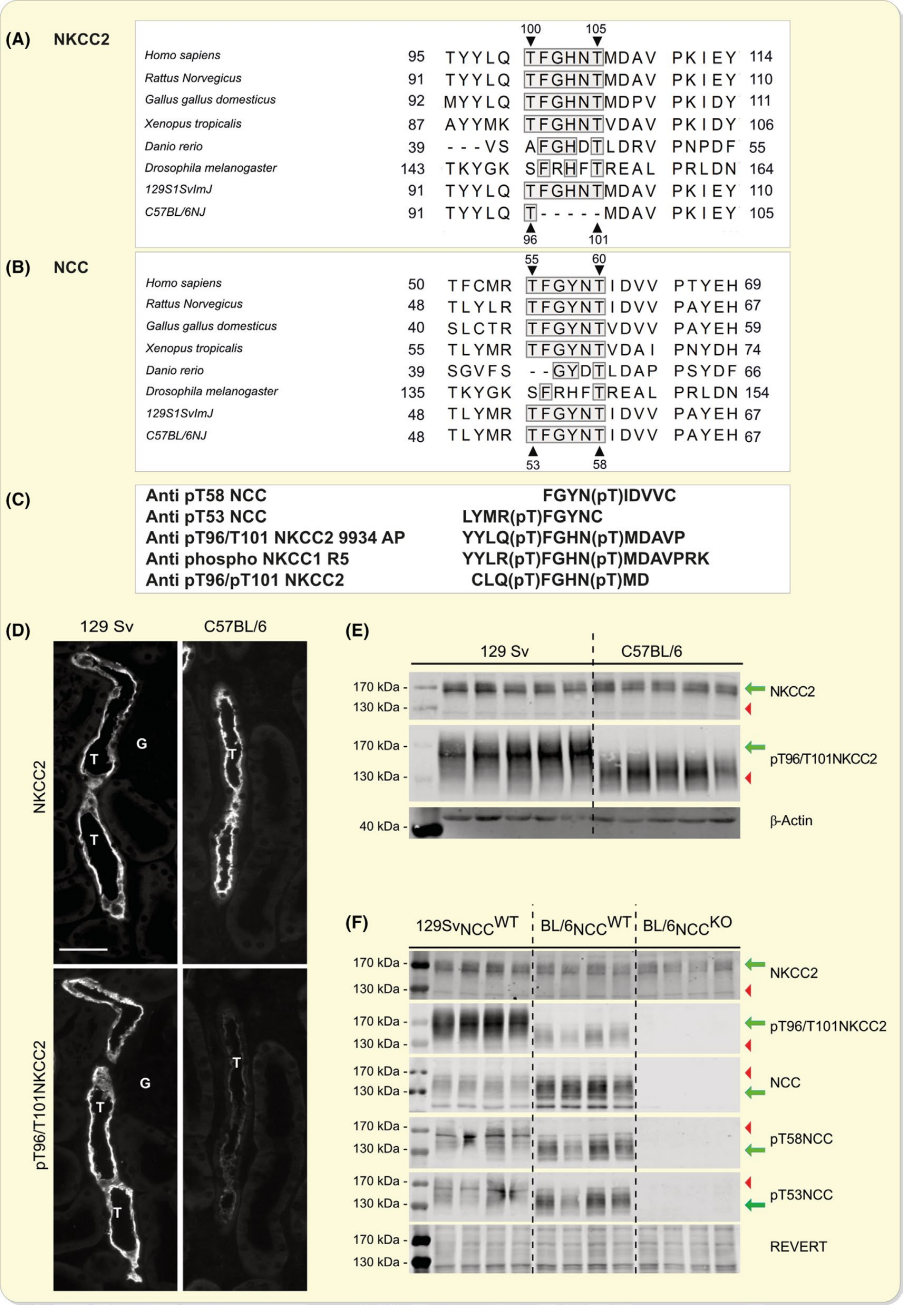
The pT96/T101 NKCC2 antibody does not detect a visible pNKCC2 signal in C57BL/6 mice. A, NKCC2 amino acids sequence alignment of different species and the two mouse strains 129Sv and C57BL/6. The NKCC2 sequence IDs for the different species are listed in Table S1. The highly conserved region corresponding to murine Thr96 to murine Thr101 is marked in grey. B, Amino acids sequence alignment of NCC in different species. The NCC sequence IDs for the different species are listed in Table S1. C, List of epitopes of commonly used and published antibodies recognizing pT53 or pT58NCC, as well as pNKCC2 at the conserved locus around Thr96 to Thr101. D, Immunostaining of total and pT96/T101 NKCC2 in consecutive cryo‐sections of mice with 129Sv and C57BL/6 background. Scale bar = 50 µm. E, Immunoblot of total and pT96/T101 NKCC2 of mice with 129Sv and C57BL/6 background. Values are means ± SEM, n = 5 mice per group, the green arrows point to the specific bands while the red arrow‐heads point to the non‐specific bands. Molecular weight markers are included. Total and pT96/T101 NKCC2 bands are expected at 170 kDa. F, Immunoblots for total and pT96/T101 NKCC2, total and pT53 and pT58 NCC in NCC WT and NCC knockout (KO) mice of different backgrounds. The green arrows point to the specific bands while the red arrow‐heads point to the non‐specific bands. Molecular weight markers are included. Total and pT58 and pT53 NCC bands are expected at 130 kDa, total and pT96/T101 NKCC2 bands are expected at 170 kDa

First, we aligned for several species the published (www.ensembl.org) N‐terminal amino acids sequences (transcription IDs in Table S1) of NKCC2 and NCC (Figure 1A,B, respectively) that correspond to the epitopes recognized by the anti‐pNCC and anti‐pNKCC antibodies (Figure 1C). The alignment confirmed the high degree of conservation of the phosphorylation sites of NKCC2 and NCC^7^ that likely explains the cross‐reactivity of the pNKCC2 and pNCC antibodies observed by us and others.^18^, ^28^, ^29^ Interestingly and consistent with our hypothesis, the alignment also revealed a so far not appreciated five amino acids deletion in the sequence of NKCC2 from C57BL/6 mice (ΔF97‐T101) (Figure 1A). Sequencing of the DNA from two different C57BL/6 mouse colonies kept at the The Jackson Laboratory in the USA or Janvier Labs in France confirmed a nucleotide deletion in exon 2 of C57BL/6 NKCC2 that corresponds to the above mentioned amino acids deletion (Figure S1, Table S2).

To characterize the impact of the five amino acids deletion on the immunodetection of total and phosphorylated NKCC2, we systematically studied kidneys of C57BL/6 and 129Sv mice by immunohistochemistry and immunoblotting. Immunofluorescence with an antibody against total NKCC2 showed a strong fluorescent signal in the apical plasma membrane of TALs of 129Sv and C57BL/6 mice. However, our previously developed pT96/T101 NKCC2 antibody showed a strong apical labelling of TALs only in 129Sv mice, while TALs of C57BL/6 mice had a very weak immunostaining (Figure 1D). Consistent with this, Western blot analysis revealed a strong band for total NKCC2 in both strains of mice, while the pT96/T101 NKCC2 antibody detected a strong band at the expected size (170 kDa) only in 129Sv mice (Figure 1E). Instead, kidneys of C57BL/6 mice revealed a band at 130 kDa that corresponds to the expected size of NCC (Figure 1E). Similar results were obtained with other antibodies previously used to study NKCC2 phosphorylation (Figure S2). The pT212/T217 NKCC1 R5 antibody^11^ showed in addition to the pNCC signal a faint signal at the expected size for pNKCC2 (Figure S2). The immunoreactive band for pNKCC2 became more intense when fresh kidney lysates were used. The use of a lysis buffer with detergents (RIPA buffer) did not improve but diminished the signal intensity (Figure S3).

To test whether our pT96/T101 NKCC2 antibody indeed cross‐reacts with phosphorylated NCC in C57BL/6 mice, we probed kidney lysates from 129Sv and C57BL/6 wild‐type mice and C57BL/6 NCC knockout mice with antibodies against total NKCC2, pT96/T101 NKCC2, total NCC, and phosphorylated NCC (pT53 and pT58 NCC) (Figure 1F). The pT96/T101 NKCC2 antibody and antibodies against total NCC and pNCC did not detect bands at 130‐170 kDa in the C57BL/6 NCC knockout mice (Figure 1F). Interestingly, the immunoblot as well as additional immunohistochemistry experiments (Figure S4) indicate that not only do the pNKCC2 antibodies cross‐react with NCC in C57BL/6 mice but our pNCC antibodies (pT53 NCC and pT58 NCC) cross‐reacts with NKCC2 in 129Sv mice at the concentrations used.

### 2.2 Urinary calcium and magnesium excretion differ between 129Sv and C57BL/6 mice

**FIGURE 2:**
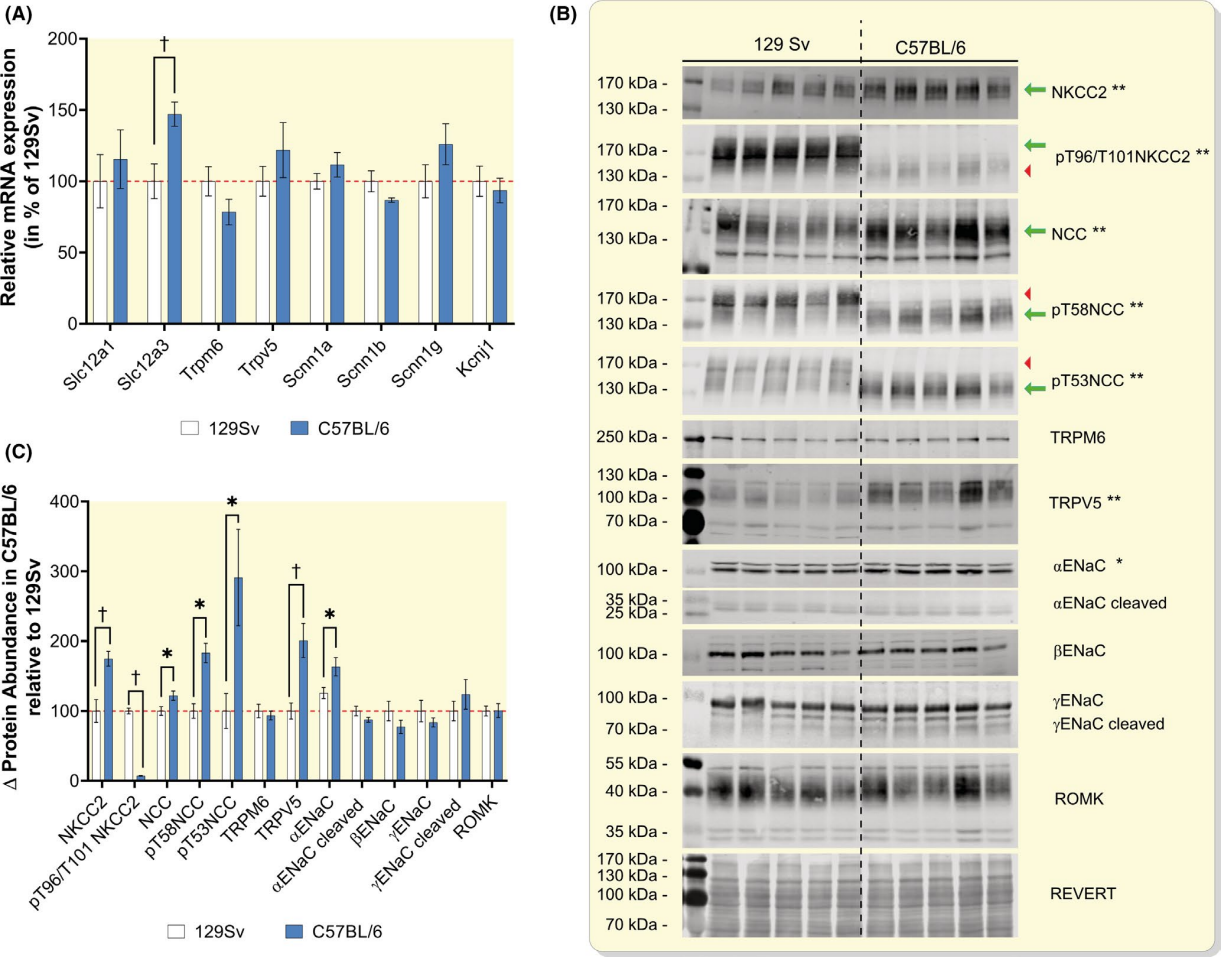
Analysis of mRNA levels and protein abundance of major renal ion transporters and channels. A, Relative mRNA expression levels of renal ion transporters and channels. Expression levels in C57BL/6 relative to those in 129Sv set as 100% (red dotted line). B, Immunoblots of major renal transporters and channels using whole kidney lysates of 129Sv and C57BL/6 mice. Molecular weight markers are included. NKCC2 and pNKCC2 are expected at 170 kDa, NCC and pNCC at 130 kDa, TRPM6 at 260 kDa, α‐ENaC at 95 kDa and 34 kDa (cleaved), β‐ENaC at 100 kDa, γ‐ENaC at 100 kDa and 85 kDa (cleaved), ROMK at 55 kDa (mature glycosylated), 43 kDa (core glycosylated) and 34 kDa (unglycosylated). Green arrows point to the specific bands while the red arrow‐heads point to the non‐specific bands. C, Densitometric analysis performed of proteins in C57BL/6 animals normalized to the average of 129Sv. The red dotted line marks the 100% of 129Sv. (Values are means ± SEM; n = 5 mice/group; *P < .05, ^†^ *P* < .01, ^‡^ *P* < .001)

To determine whether the deletion (F97‐T101) in NKCC2 may associate with physiological differences between 129Sv and C57BL/6 mice, we compared water intake, plasma ion levels, and urinary fluid and ion excretion between the two mouse strains. As summarized in Table 1, most of the parameters assessed did not differ between the strains. However, C57BL/6 mice showed a lower urinary Ca^2+^ and higher urinary Mg^2+^ excretion than 129Sv mice. The plasma Ca^2+^ and Mg^2+^ levels were similar for both groups, suggesting that the mice were in a steady‐state at which a homeostatic balance of intestinal reabsorption, tissue mobilization (eg from bone), and renal excretion of Ca^2+^ and Mg^2+^ ions was reached. To test whether the different urinary Ca^2+^ and Mg^2+^ excretion rates result from differences in the intestinal up‐take of these ions, we compared daily food intake, faecal output, and faecal Ca^2+^ and Mg^2+^ excretion for the two mouse strains. As shown in Figure S5, food intake and faecal Ca^2+^ output were similar, but the daily faecal Mg^2+^ excretion was lower in C57BL/6 mice than in 129Sv mice suggesting that C57BL/6 mice have higher intestinal Mg^2+^ reabsorption than 129Sv mice. Consistent with this assumption, C57BL/6 mice had stronger colonic expression of the magnesium channel TRPM6 (Figure S6), as well as a trend to higher plasma Mg^2+^ levels compared to 129Sv mice, though the differences did not reach statistical significance. To assess the possible contribution of the bone, we analysed by micro‐CT the bone mineral density in both strains. Similar to previous reports,^32^ C57BL/6 mice had a rather low bone mineral density (Figure S7), which may explain why these mice tend to conserve Ca^2+^ ions via a reduced renal Ca^2+^ excretion. Consistent with an enhanced tubular Ca^2+^ reabsorption rate, the kidneys of C57BL/6 mice expressed more TRPV5 Ca^2+^ channel at protein levels than kidneys of 129Sv mice (Figure 2). C57BL/6 mice had also a significantly greater NCC mRNA and protein expression than 129Sv mice (Figure 2A). Likewise, the protein abundance of total NKCC2, total and phosphorylated NCC and of total α‐ENaC were higher in C57BL/6 than in 129Sv mice (Figure 2B,C). The abundances of the proteolytically cleaved and hence active forms of α‐ and γ‐ENaC were not different suggesting that the renal ENaC activity was similar for both strains (Figure 2B,C).

**TABLE 1:** Physiological parameters for renal function between C57BL/6 mice and 129Sv mice

Standard diet (0.2% Na^+^, 0.78% K^+^)					
	129Sv	n	C57BL/6	n	*P*
Physiological parameters					
Body weight [g]	25.8 ± 0.5	11	26.3 ± 0.3	11	ns
Heart weight [g]	0.2 ± 0.0	6	0.2 ± 0.0	5	ns
Water intake [g/24 h]	3.7 ± 0.3	11	4.6 ± 0.2	11	*
Urinary parameters					
Urine volume [µL/24 h]	1.4 ± 0.1	11	1.7 ± 0.1	11	ns
Urine osmolality [mOsm/kgH_2_O]	2131 ± 195	11	2229 ± 292	11	ns
Urine osmolality [mOsm/kgH_2_O] with H_2_O restriction	2656 ± 160	11	2903 ± 157	11	ns
Urinary creatinine [µmol/24 h]	3.8 ± 4.2	11	4.2 ± 0.5	11	ns
UMOD [mg/24 h/g Creatinine]	31.8 ± 5.7	11	34.9 ± 4.7	11	ns
UNa^+^ /UVolume [µmol/24 h]	133.9 ± 19.8	11	157.9 ± 14.2	11	ns
UK^+^ /UVolume [µmol/24 h]	439.4 ± 57.8	11	453.9 ± 33.3	11	ns
UCa^2+^/UVolume [µmol/24 h]	5.3 ± 0.5	11	1.5 ± 0.3	11	^‡^
UMg^2+^/UVolume [µmol/24 h]	15.4 ± 2.9	11	28.5 ± 3.3	11	^†^
Urinary phosphate [µmol/24 h]	0.7 ± 0.1	6	1.0 ± 0.1	6	ns
Plasma parameters					
Na^+^ [mmol/L]	158.2 ± 1.8	4	161.1 ± 2.0	5	ns
K^+^ [mmol/L]	3.6 ± 0.1	4	3.9 ± 0.1	5	ns
Ca^2+^ [mmol/L]	2.32 ± 0.04	10	2.26 ± 0.05	9	ns
Mg^2+^ [mmol/L]	1.4 ± 0.2	11	1.9 ± 0.3	10	ns
Phosphate [mmol/L]	1.6 ± 0.1	10	1.7 ± 0.1	7	ns
Aldosterone [nmol/L]	0.79 ± 0.1	5	1.1 ± 0.2	5	ns

Note

Urinary and serum parameters of 129Sv and C57BL/6 mice. Urinary ion excretion over 24 h was normalized to the urinary creatinine concentration. Values are means ± SEM.

* *P* < .05,

^†^ *P* < .001,

^‡^ *P* < .0001.

### 2.3 No evidence for an altered thick ascending limb function in C57BL/6 mice compared with 129Sv mice

**FIGURE 3:**
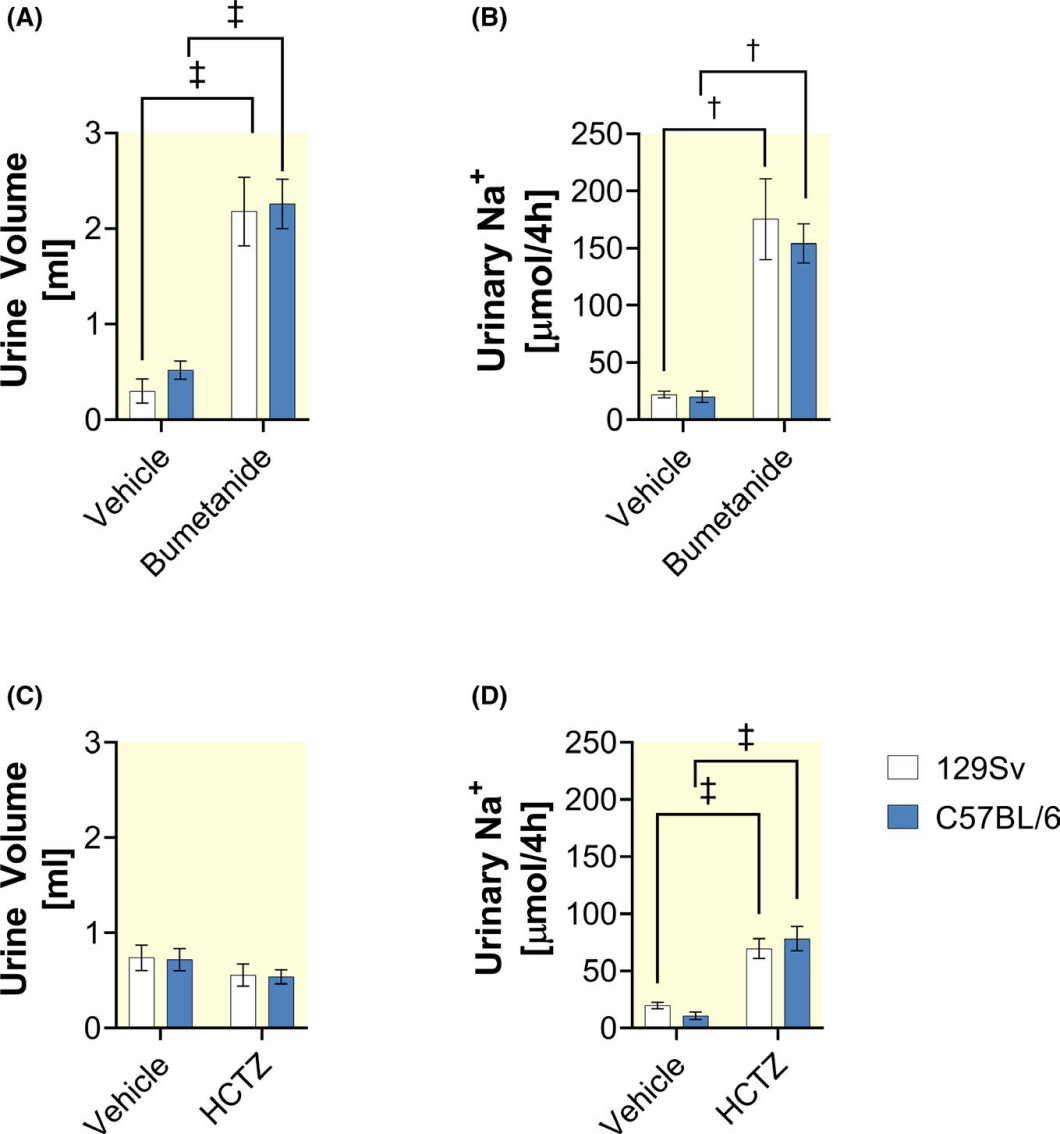
Bumetanide and thiazide tests give similar results for C57BL/6 and 129Sv mice. A,B, Urine volumes and urinary Na^+^ excretions in 129Sv and C57Bl/6 mice within 4 h after a single i.p. injection of either vehicle or bumetanide. C,D, Urine volumes and urinary Na^+^ excretions in 129Sv and C57Bl/6 mice within 4 h after a single i.p. injection of either vehicle or bumetanide. (Values are means ± SEM; n = 5 mice/group; *P < .05, ^†^ *P* < .01, ^‡^ *P* < .001)

The higher abundances of total NKCC2, total NCC and phosphorylated NCC in kidneys of C57BL/6 mice may be a compensatory response for reduced NKCC2 phosphorylation. To test whether the activity of NKCC2 and NCC differs between the mouse strains, we performed a bumetanide‐ and thiazide‐test respectively. A single injection of bumetanide or hydrochlorothiazide provoked a strong natriuresis which was more pronounced for bumetanide than for hydrochlorothiazide. However, the diuretic responses were completely similar in both strains suggesting that the functional activities of NKCC2 and NCC were not different between C57BL/6 and 129Sv mice (Figure 3A‐D). Apparently, protein abundances and phosphorylation levels do not necessarily match with the functional activities of renal ion transport proteins as recently also pointed out by Hunter and co‐workers.^33^ To further assess TAL function, we performed a water restriction test in which mice had for 24 hours access to wetted food, but not to tap water. Under these conditions, both mouse strains increased urine osmolarity to similar values (Table 1). Furthermore, C57BL/6 mice and 129Sv mice showed the same urinary excretion of uromodulin (Tamm‐Horsfall protein) (Table 1), which is produced and excreted into the urine depending on the functional activity of the TAL cells.^34^

### 2.4 Crossbreeding of 129Sv and C57BL/6 mice suggests that the five amino acids deletion in NKCC2 does not segregate a specific phenotype

**FIGURE 4:**
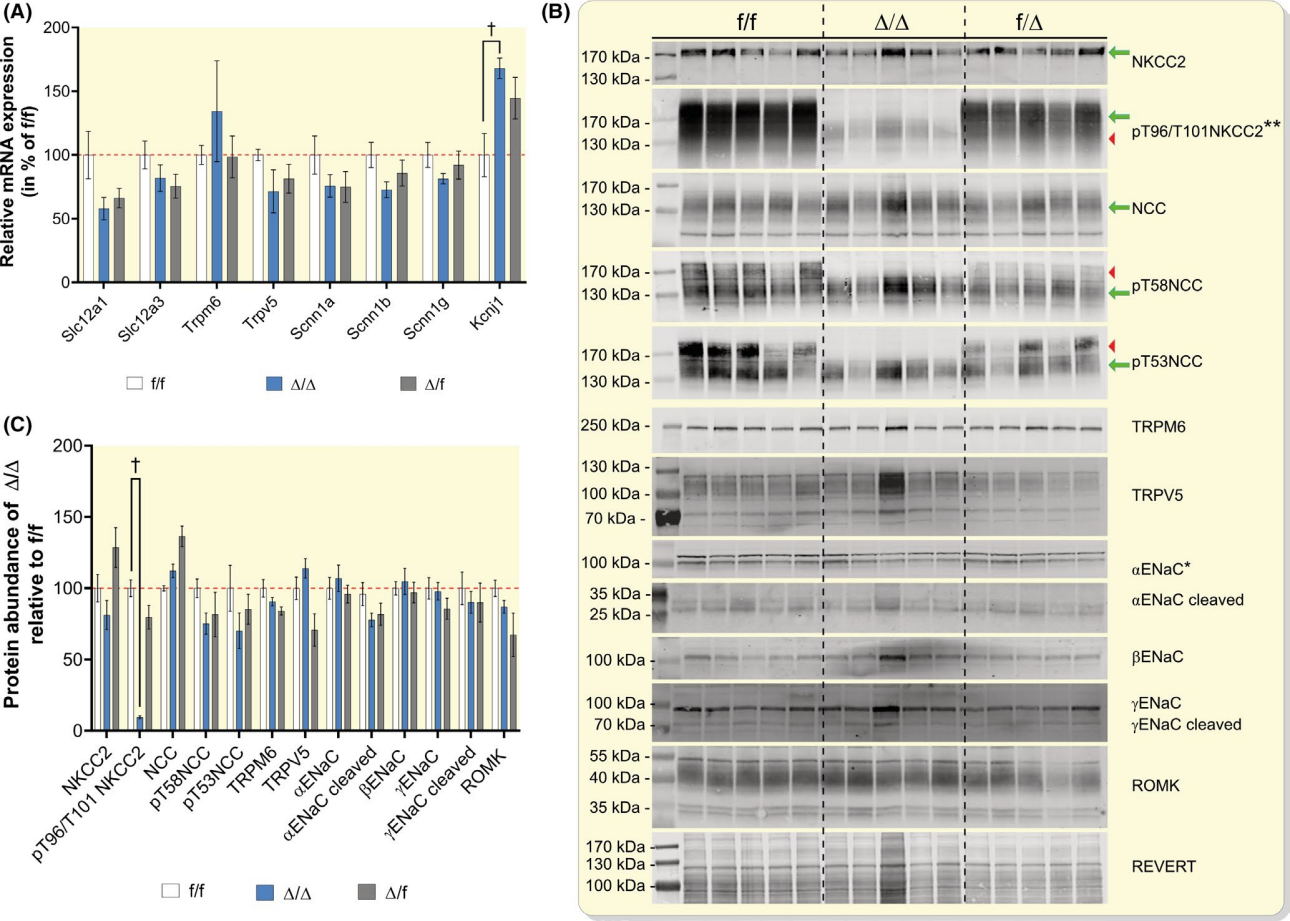
No significant changes in mRNA and protein expression of major renal ion transporters and channels in the crossbred F2 generation. A, Bar plot of relative mRNA expression levels in the three groups of mice. All values were normalized to f/f mice. Values are means ± SEM. B, Protein abundance in mouse kidney lysates from animals of the F2 generation. The three groups correspond to full‐length NKCC2 (f/f), NKCC2 homozygous for the deletion (Δ/Δ) or NKCC2 heterozygous for the deletion (f/Δ/). Molecular weight markers are included. NKCC2 and pNKCC2 are expected at 170 kDa, NCC and pNCC at 130 kDa, TRPM6 at 260 kDa, α‐ENaC at 95 kDa and 34 kDa (cleaved), β‐ENaC at 100 kDa, γ‐ENaC at 100 kDa and 85 kDa (cleaved), ROMK at 55 kDa (mature glycosylated), 43 kDa (core glycosylated) and 34 kDa (unglycosylated). Green arrows point to the specific bands while the red arrow‐heads point to the non‐specific bands. C, Densitometric analysis of protein abundance comparing f/f and Δ/Δ mice. The band intensities from Δ/Δ mice are normalized to those from f/f. (Values are means ± SEM; n = 5 mice/group; *P < .05, ^†^ *P* < .01, ^‡^ *P* < .001)

The presented data suggest that the strain differences in urinary ion excretions and renal protein abundances are not related to the five amino acids deletion in C57BL/6 NKCC2, but are related to other genetic differences between the two mouse strains. To test this further, we crossbred 129Sv and C57BL/6 mice to the F2 generation in which genetic strain differences (including those in NKCC2) should be randomly distributed to the mice. We then compared F2 mice that were either homozygous for full‐length NKCC2 (f/f), homozygous for the NKCC2 deletion (Δ/Δ), or heterozygous for the NKCC2 deletion (f/Δ). With the exception of the expected different binding patterns of the phosphoform‐specific antibodies against NKCC2 and NCC (and an unclear difference of ROMK expression at the mRNA, but not protein level), none of the previously identified differences between the two strains were evident in the f/f, Δ/Δ, or f/Δ mice of the F2 generation (Figure 4 and Table 2). In particular, there were no differences in the urinary Ca^2+^ or Mg^2+^ excretion, as well as no differences in plasma Ca^2+^ or Mg^2+^ levels, suggesting that the strain differences between 129Sv and C57BL/6 mice are independent of the five amino acids deletion in NKCC2.

**TABLE 2:** Crossbred F2 mice are phenotypically similar regardless of the genotypes

Standard diet (0.2% Na^+^, 0.78% K^+^)					
	f/f	n	Δ/Δ	N	*P*
Physiological parameters					
Body weight [g]	26 ± 0.6	5	26.9 ± 0.4	5	ns
Heart weight [g]	ND		ND		
Food intake [g/24 h]	4.1 ± 0.3	5	3.8 ± 0.5	5	ns
Water intake [g/24 h]	4.2 ± 0.2	5	4.9 ± 0.6	5	ns
Faecal output [g/24 h]	0.26 ± 0.03	5	0.20 ± 0.04	5	ns
Urinary parameters					
Urine volume [µL/24 h]	1.7 ± 0.1	5	1.5 ± 0.1	5	ns
Urine osmolality [mOsm/kgH_2_O]	2123 ± 62	5	2285 ± 235	5	ns
Urine osmolality [mOsm/kgH_2_O] with H_2_O restriction	ND		ND		ND
Urinary creatinine [µmol/24 h]	2.2 ± 0.4	5	2.6 ± 0.5	5	ns
UMOD [mg/24 h/g creatinine]	56.2 ± 10.1	5	77.9 ± 16.0	5	ns
UNa^+^ /UVolume [µmol/24 h]	117.8 ± 10.1	5	108.5 ± 8.1	5	ns
UK^+^ /UVolume [µmol/24 h]	391.5 ± 19.3	5	348.2 ± 38.5	5	ns
UCa^2+^/UVolume [µmol/24 h]	0.7 ± 0.1	5	0.6 ± 0.2	5	ns
UMg^2+^/UVolume [µmol/24 h]	5.9 ± 0.9	5	7.9 ± 2.1	5	ns
Plasma parameters					
Na^+^ [mmol/L]	150.2 ± 1.4	5	148.2 ± 1.2	5	ns
K^+^ [mmol/L]	3.4 ± 0.1	5	385 ± 0.2	5	ns
Ca^2+^ [mmol/L]	2.2 ± 0.1	5	2.2 ± 0.1	5	ns
Mg^2+^ [mmol/L]	2.1 ± 0.1	5	2.2 ± 0.1	4	ns
Phosphate [mmol/L]	1.8 ± 0.1	5	1.8 ± 0.1	3	ns
Aldosterone	ND		ND		

Note

Urinary and serum parameters in F2 crossbred mice with the hybrid strain 129S/B6. Values are means ± SEM.

Abbreviation: ND, not determined.

* *P* < .05,

^†^ *P* < .001,

^‡^ *P* < .0001.

### 2.5 A novel pT96 NKCC2 antibody detects pNKCC2 in both strains

**FIGURE 5:**
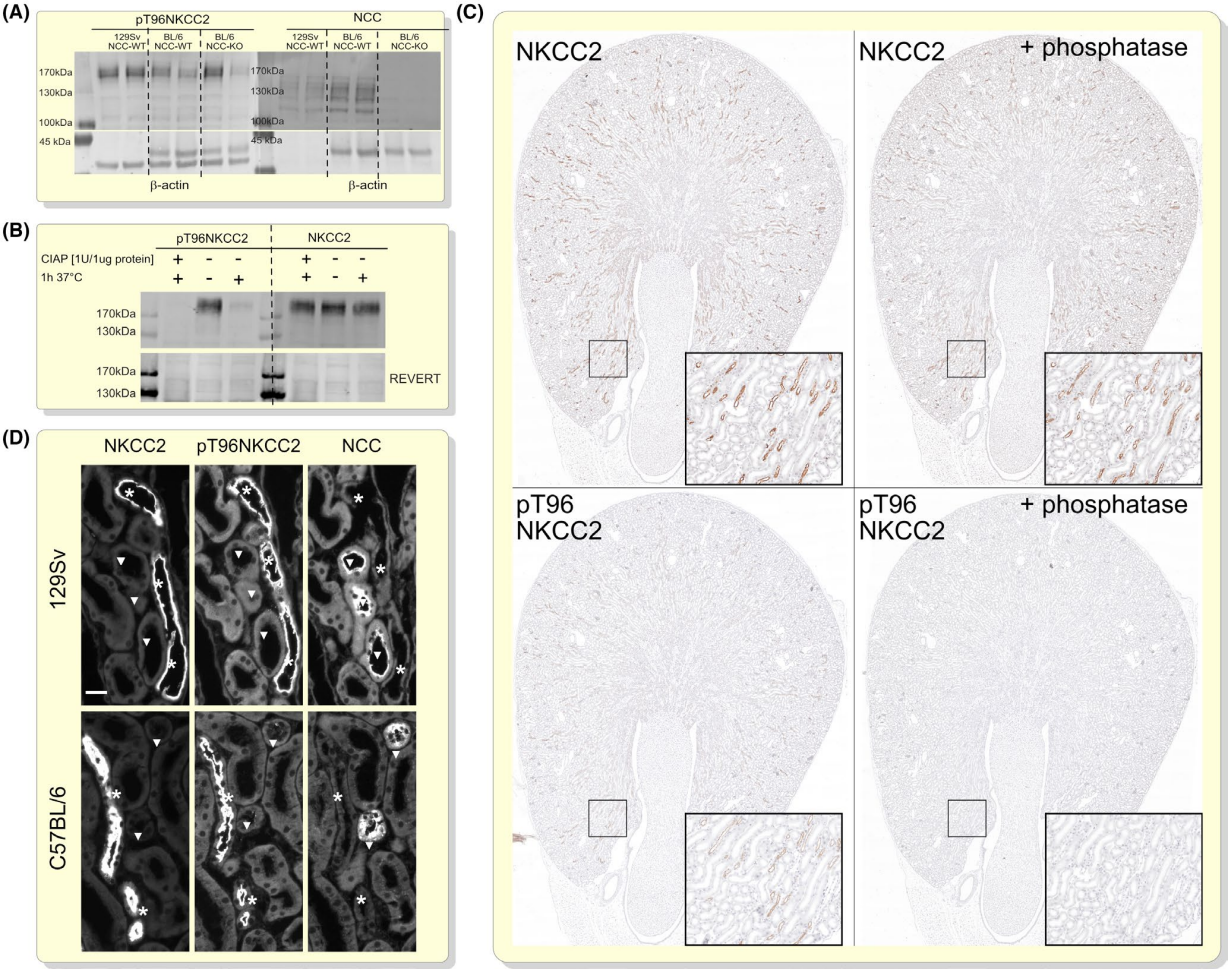
A new pT96 NKCC2 antibody allows detection of pNKCC2 in both strains. A, Immunoblot of whole kidney protein lysates of 129Sv and C57BL/6 mice and C57BL/6 NCC‐KO mice. β‐actin was used as loading control. Molecular weight markers are included, NKCC2 and pNKCC2 are expected at 170 kDa, NCC at 130 kDa. B, Dephosphorylation assay using calf intestine alkaline phosphatase (CIAP) on whole kidney lysates of 129Sv lysates without phosphatase inhibitors. One half of the membrane was incubated with pT96 NKCC2, while the second half was incubated with tNKCC2 antibody. Molecular weight markers are included, pNKCC2 is expected at 170 kDa. C, Immunostaining on whole kidney consecutive paraffin sections from C57BL/6 mice. Sections were incubated ± lambda phosphatase and probed against NKCC2 and pT96 NKCC2. D, Immunostaining on cryo‐sections of 129Sv and C57BL/6 mice. Sections were stained with tNKCC2 in order to identify TALs, NCC staining was used for DCT identification. T = thick ascending limb (TAL), D = distal convoluted tubule (DCT). Bar = 25µm

**FIGURE 6:**
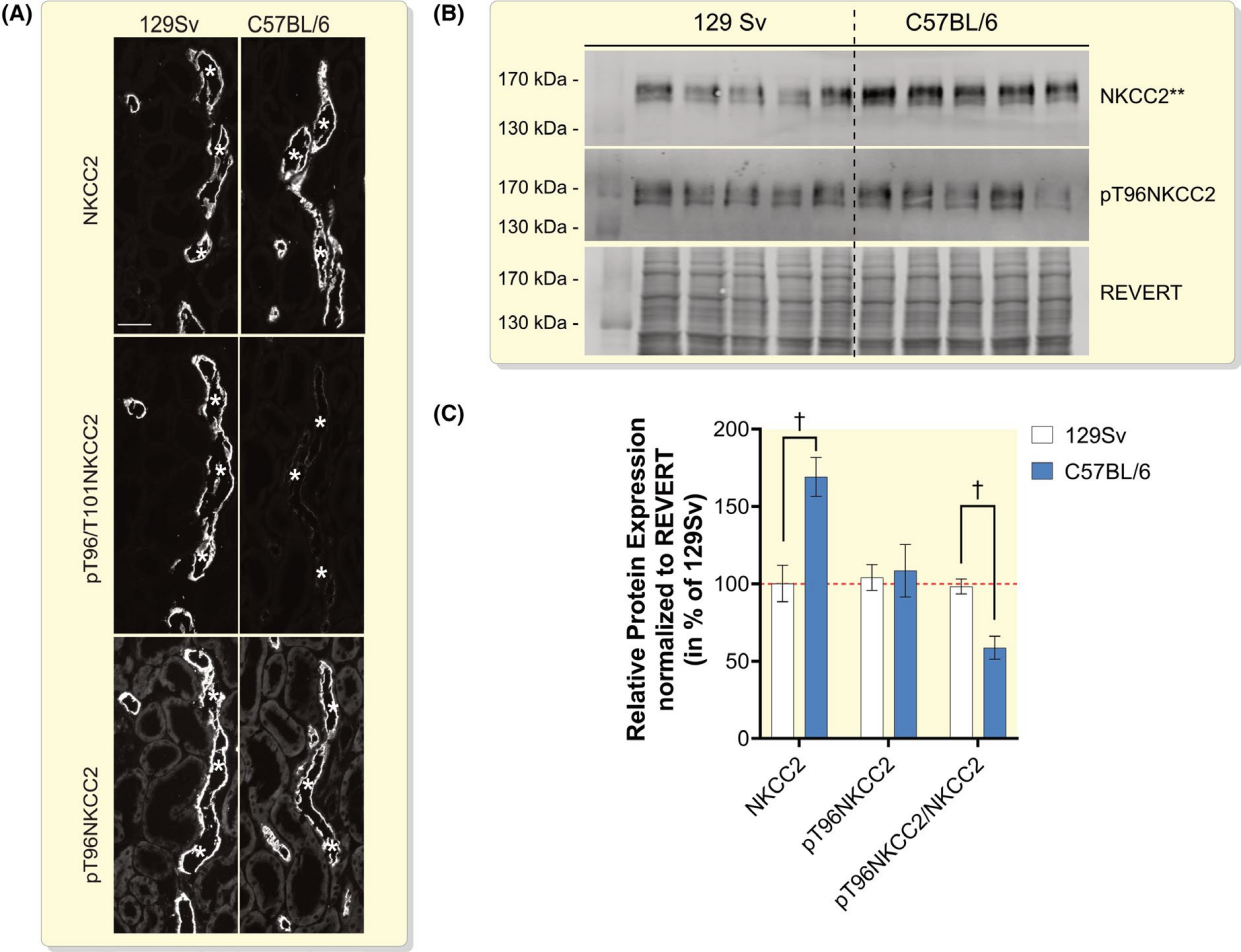
A new pT96 NKCC2 antibody detects equally well pNKCC2 abundance in 129Sv and C57BL/6 mice. A, Immunostaining of total, pT96/T101 and pT96 NKCC2 in consecutive cryo‐sections (5 µm) of mice with 129Sv and C57BL/6 background. Bars =50 µm, *TAL. B, Immunoblot of total and pT96 NKCC2 of mice with 129Sv and C57BL/6 background. Molecular weight markers are included. The expected band size for total and pT96 NKCC2 is 170 kDa. Revert Total Protein stain was used as loading control. (Values are means ± SEM; n = 5 mice/group; *P < .05, ^†^ *P* < .01, ^‡^ *P* < .001)

Since the five amino acids deletion in NKCC2 hinders a reliable detection of NKCC2 phosphorylation in C57BL/6 mice when using available antibodies, we generated a new antibody directed against the T96 phospho‐site in C57BL/6 mice. Rabbits were immunized with a phospho‐peptide that corresponded to the amino acid sequence of C57BL/6 NKCC2 surrounding T96 (YYLQ(p)TMDA). The antisera were affinity‐purified and characterized by immunoblotting and immunohistochemistry (Figure 5). The antibody detected a single band at the expected size of 170 kDa in kidneys of 129Sv, C57BL/6 wild‐type and C57BL/6 NCC knock‐out mice (Figure 5A). The band was clearly different from the one detected for NCC at 130 kDa. Dephosphorylation of 129Sv kidney samples with calf intestine alkaline phosphatase (CIAP) completely eradicated the signal obtained with the new pT96 NKCC2 antibody, while the signal with the non‐phosphoform‐specific NKCC2 antibody was visible in control and dephosphorylated samples (Figure 5B). Interestingly, NKCC2 phosphorylation was also largely eliminated when kidney lysates were incubated at 37℃ for 1 hour without CIAP (likely because of activity of endogenous alkaline phosphatase from the proximal tubules). Dephosphorylation experiments using lambda phosphatase on consecutive paraffin‐kidney sections of C57BL/6 mice further confirmed that the new antibody recognizes only phosphorylated NKCC2 (Figure 5C). Moreover, immunofluorescence on consecutive cryosections demonstrated that new the antibody stains only the apical plasma membrane of NKCC2‐positive TALs but not of NCC‐positive DCTs in 129Sv and C57BL/6 mice (Figure 5D), suggesting that the new pNKCC2 antibody does not cross‐react with pNCC. This conclusion was also confirmed using cell lysates from MDCKI cells expressing human NKCC2 (hNKCC2) and human NCC (hNCC). Immunoblots of immunoprecipitations (IP) and whole cell lysates demonstrated that the novel pT96 NKCC2 antibody recognizes human NKCC2 but not human NCC (Figure S8A). Interestingly, although the new antibody was raised against pNKCC2 of C57BL/6 mice, the new antibody detected NKCC2 phosphorylation equivalently in kidneys of both mouse strains by both immunohistochemistry (Figure 6A) and immunoblotting (Figure 6B).

### 2.6 The novel pT96 NKCC2 antibody permits analysis of regulated NKCC2 phosphorylation

**FIGURE 7:**
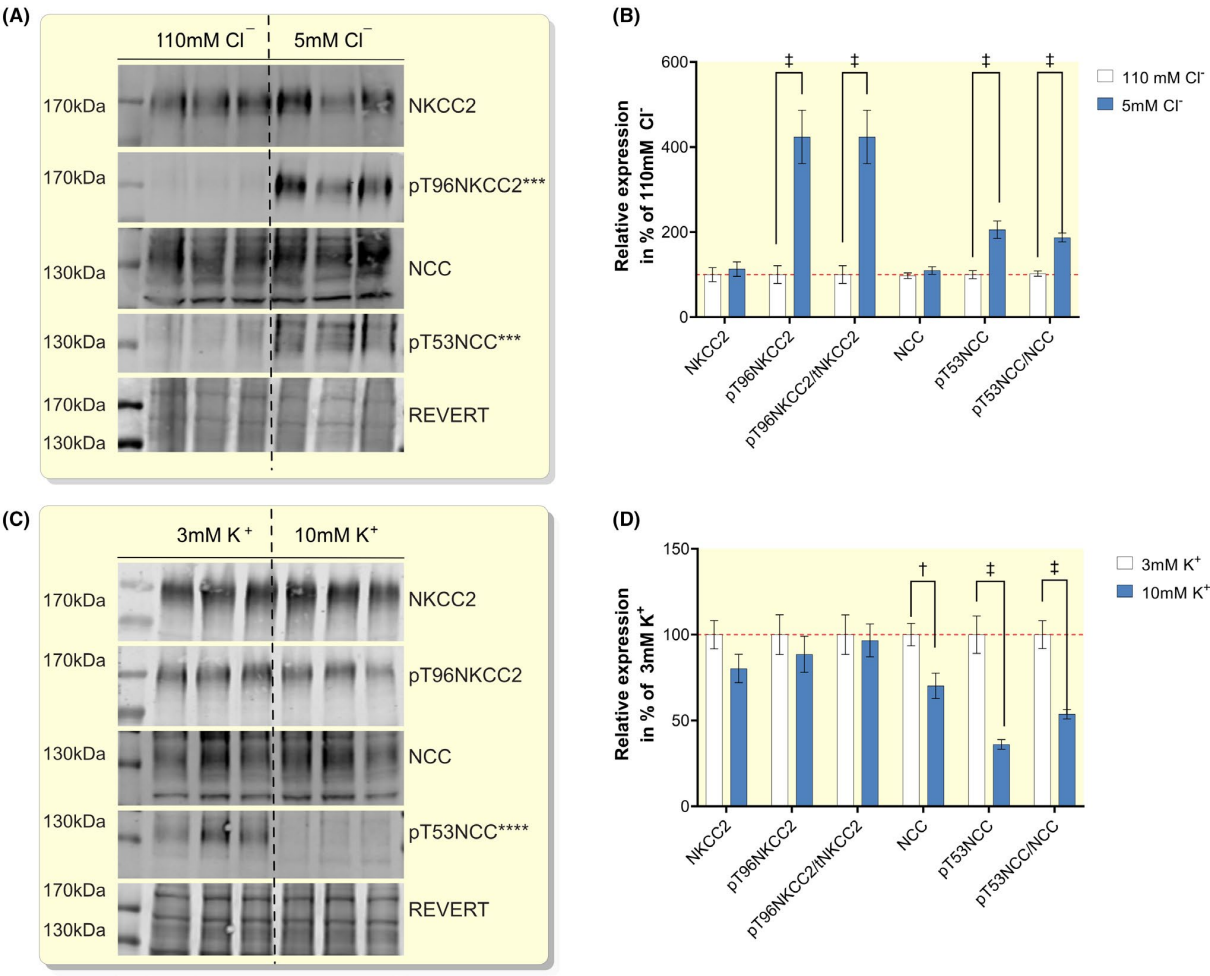
Regulation of NKCC2 phosphorylation detected by the newly generated antibody. A, Immunoblots of protein lysates of kidney slices (50 µg of protein) incubated in control (110 mM) as well as low (5 mM) [Cl^−^]_ex_. Immunoblots were probed against NKCC2 and pT96 NKCC2, as well as NCC and pT53 NCC. Revert total protein stain has been used as loading control. B, Densitometric analysis of immunoblots for NKCC2 and pT9 6NKCC2 in 7a. C, Immunoblot of kidney slices (50 µg of protein per kidney slice) protein lysates incubated in control (3 mM) as well as high (10 mM) [K^+^]_ex_. Immunoblots were probed against NKCC2 and pT96 NKCC2, as well as against NCC and pT53NCC. Revert total protein stain was used as loading control. D, Densitometric analysis of NKCC2 and pT96 NKCC2 in 7c. Protein abundance of kidney slices treated with 3 mM K^+^ was set 100% for normalization. Values are means ± SEM; n = 9 (three kidney slices from each mouse: total three mice per group)

The phosphorylation and hence the activity of NKCC2 and NCC are regulated by extracellular ion concentrations. A low [Cl^−^]_ex_ increases the phosphorylation of NKCC2 at Thr96 and Thr101 and of NCC at Thr53 and Thr58 via activation of the WNK/SPAK/OSR1 pathway.^7^, ^8^, ^18^, ^29^, ^35^ Likewise, a high [K^+^]_ex_ rapidly decreases the phosphorylation of NCC, but is thought to have little or no effect on NKCC2 phosphorylation.^12^, ^36^, ^37^, ^38^ To test whether the newly developed pT96 NKCC2 antibody is able to detect this type of differential regulation of NKCC2 phosphorylation, kidney slices of C57BL/6 mice were exposed ex vivo to either 110 or 5 mM [Cl^−^]_ex_ and to 3 or 10 mM [K^+^]_ex_ respectively. As expected, the low [Cl^−^]_ex_ strongly increased NCC and NKCC2 phosphorylation (Figure 7A,B), while the high [K^+^]_ex_ only decreased NCC phosphorylation but did not alter NKCC2 phosphorylation (Figure 7C,D). Likewise, the new antibody detected equally well altered NKCC2 phosphorylation that was provoked in MDCKI cells expressing human NKCC2 (hNKCC2) or mouse NKCC2 (mNKCC2(C57BL/6)) by hypotonic low extracellular chloride conditions (Figure S8B). In kidney slices incubated at 5 mM [Cl^−^]_ex_, also the previously described anti‐mouse pT96/T101 NKCC2^39^ and anti‐human pT212/T217 NKCC1 R5^11^ antibodies detect faint signals for NKCC2 and NCC, which get more obvious when more proteins are loaded and immunoblots are imaged at enhanced settings (Figure S9).

## 3 DISCUSSION

C57BL/6 and 129Sv mice are two of the most commonly used mouse strains for biomedical research. The genome of C57BL/6 mice was the first that was sequenced for the mouse^40^ and has served since as the reference genome for the analysis of genetic and phenotypic variations between mouse strains.^41^, ^42^, ^43^, ^44^ For kidney research, one essential difference between C57BL/6 and 129Sv mice concerns the expression of the renin gene. Like humans, C57BL/6 mice harbour only one renin gene, while the 129Sv mice possess two copies, which may explain the higher susceptibility of this mouse strain to hypertension^45^, ^46^ and hypertensive injury.^47^ With the present study, we add another important genetic difference that needs to be considered. We describe a hitherto unappreciated five amino acids deletion in NKCC2 (ΔF97‐T101), present in C57BL/6 but not in 129Sv mice, that interferes with the detection of NKCC2 phosphorylation with most previously available antibodies, but does not significantly impact kidney function.

The mouse NKCC2 N‐terminal phosphorylation sites T96 and T101 are highly conserved across species and between NKCC2, NKCC1 and NCC (Figure 1).^48^ Therefore, it is not surprising that antibodies directed against these phosphorylation sites cross‐react with the other cotransporters. Previous studies have reported that antibodies against phosphorylated NCC also detect phosphorylated NKCC2^7^, ^18^, ^28^, ^29^ and that a pNKCC1 antibody allows analysis of the phosphorylation levels of both NKCC1 and NKCC2.^11^ Consistent with this, we observed cross‐reactivity of our pNCC (pT53 and pT58) antibodies with phosphorylated NKCC2 and of our pT96/pT101 NKCC2 antibody with phosphorylated NCC. However, the cross‐reactivity of the pNCC antibodies with NKCC2 was seen only in kidneys of 129Sv mice, but not in kidneys of C57BL/6 mice. Conversely, our previously developed antibody to NKCC2 pT96/pT101 showed no or at best a very weak signal for pNKCC2 in the kidneys of C57BL/6 mice and detected mainly phosphorylated NCC in this strain. Now, we link these confusing observations to a five amino acids deletion in NKCC2 of C57BL6 mice (ΔF97‐T101).

The regulation of NKCC2 phosphorylation has been studied in various experimental settings and for various stimuli including vasopressin,^14^, ^21^, ^24^, ^25^, ^49^ uromodulin^19^, ^20^, ^50^ and calcineurin inhibitors.^10^, ^26^ While most of the studies were performed in heterologous expression systems^10^, ^19^, ^20^, ^25^, ^50^ or in rats^10^, ^24^ and mice^19^, ^26^ expressing full length NKCC2, some studies were also performed in C57BL/6 mice.^7^, ^18^, ^27^, ^28^, ^29^, ^51^ As we report here, C57BL/6 mice express a NKCC2 variant with a five amino acids deletion that interferes with the proper detection of NKCC2 phosphorylation and that bears the risk that standard pT96/T101 NKCC2 antibodies and the R5 anti‐phospho‐NKCC1 antibody cross‐react with phosphorylated NCC at least under the conditions used in our work. It is not possible to judge the impact of this cross‐reaction on previous experimental conclusions, but according to our observations, data from C57BL/6 mice should be revisited and interpreted with caution.

Despite the fact, that the five amino acids deletion had a strong impact on the detection of NKCC2 phosphorylation, we have no evidence that the deletion significantly affects the functional activity of the cotransporter. C57BL/6 mice had indeed a higher urinary Mg^2+^ excretion and lower urinary Ca^2+^ excretion^31^ than 129Sv mice, but we found that these differences are probably not related to an altered paracellular transport of these cations along the TAL, but are the result of an enhanced TRPM6‐dependent Mg^2+^ uptake in the intestine and an increased TRPV5‐dependent Ca^2+^ reabsorption in DCT2 and CNT. The latter causes a positive Ca^2+^ balance that may aid to increase the low bone mineral density of C57BL/6 mice. The finding that C57BL/6 and 129Sv mice show the same renal responses to loop‐ and thiazide diuretics and to water restriction further suggests that the five amino acids deletion in NKCC2 does not significantly impair TAL function. Most importantly, mice of the F2 generation of cross‐bred C57BL/6 and 129Sv mice have the same renal phenotype independent of whether mice express full length (f/f) NKCC2 or NKCC2 homozygous (Δ/Δ) for the deletion. These observations in mice are consistent with previous functional data from heterologous expression systems. While mutations of single residues in NKCC1 (T217 in human, T211 in mouse) or NCC (T60 in human, T58 in mouse) abolish the activity of these cotransporters, mutations of the corresponding residue in NKCC2 (T105 in human, T101 in mouse) have rather little or even no effect on baseline and stimulated NKCC2 activity.^35^, ^52^, ^53^ Even when both threonine residues in NKCC2 are mutated to alanine, the functional activity of NKCC2 still reaches ~70% of maximum activity,^35^ suggesting that the phosphorylation of these sites is indicative but not essential for NKCC2 activity.

Phosphorylation sites are often homologous between proteins. Hence, the problem of cross‐reactivity of phosphoform‐specific antibodies is not unique for antibodies directed against phosphorylated NCC, NKCC2 and NKCC1, but was reported also for other proteins such as the cyclin dependent kinases 1 and 2^54^ and the extracellular signal‐regulated kinases 1 and 2.^55^ Consistently, whenever phosphorylation state‐specific antibodies are used, rigorous experimental controls are needed to confirm the specificity of the detected signals. This may include (a) confirmation that the employed antibody detects the protein at the expected molecular weight (immunoblotting) and/or at the expected cellular and subcellular localization (immunofluorescence), (b) verification that the antibody interacts specifically with the phosphorylated epitope by using competition assays with the phosphorylated and non‐phosphorylated peptides, and (c) demonstration that the antibody is phosphoform specific by comparing phosphorylated and dephosphorylated tissue samples. These controls might be complemented by genetic controls that include the testing of tissues from knockout animals and/or cells heterologously expressing the protein of interest.

In the present study, we used above‐mentioned approaches to thoroughly characterize a new pT96 NKCC2 antibody that detects phosphorylated NKCC2 in kidney samples from C57BL/6 and 129Sv mice equally well. The antibody is phosphoform‐specific and shows no apparent cross‐reactivity with phosphorylated NCC under any of the tested conditions. Using this antibody we confirmed that low extracellular chloride concentrations increase the phosphorylation of NKCC2 at this site,^7^, ^8^ while altered extracellular potassium concentrations do not have a significant regulatory role.^24^

In conclusion, our work reveals a critical strain difference in the amino acids sequence of mouse NKCC2 that impacts the detection and hence the analysis of NKCC2 phosphorylation and regulation. A newly developed pT96 NKCC2 antibody circumvents this technical caveat and allows a reliable detection of altered NKCC2 phosphorylation independent from the genetic background of mouse models. Moreover, our study emphasizes again that genetic background differences have to be considered when analysing mouse models.

## 4 MATERIALS AND METHODS

### 4.1 Animals

Male age matched (6‐8 weeks) mice were either of inbred strains 129S6/SvEvTac, C57BL/6J, hereby after referred to as 129Sv and C57BL/6, or a mixed hybrid strain 129S/B6 in F2 generation. All animal experiments were conducted according to Swiss Animal Welfare laws and approved by the veterinary administration of the Canton of Zurich, Switzerland (Licenses: 141/2014, 185/2017 and 135/2018). Mice were genotyped for the deletion of 15 bases using standard PCR with primers provided in Table S3. Full‐length (f/f) NKCC2 has an expected PCR product of 87 bp while deleted NKCC2 sequence (Δ/Δ) is expected at 70 bp.

### 4.2 Tissue processing

Mice were anaesthetized with a combination of isoflurane (2%‐4%, 0.5 l/min; Provet; CH) and Temgesic (Buprenorphine; 0.05‐0.4 mg/kg body weight; Indivor; CH). For biochemical analysis, mice were anaesthetized and perfused via the left ventricle of the heart with phosphate buffered saline (PBS). Kidneys and distal colons were harvested, snap frozen in liquid nitrogen and stored in −80℃ until further processing. For immunohistochemistry, kidneys were fixed using paraformaldehyde (PFA) 3% in 0.1 M phosphate buffer (pH 7.4, 300 mOsm) by retrograde perfusion of the deeply anaesthetized mouse via the abdominal aorta followed by a short rinsing with 0.1 M phosphate buffer (0.2 M NaH_2_PO_4_ x H_2_O, 0.2 M NaH_2_PO_4_ x 2H_2_O, 0.1 M CaCl_2_). Subsequently, kidneys were removed, cut into pieces and frozen in liquid propane and stored at −80℃.

### 4.3 Metabolic cage studies

After adaption to metabolic cages (Techniplast), mice were kept in the metabolic cages for four consecutive days with free access to tap water and standard lab chow (Ssniff, Spezialdiäten GmbH). Body weight, food and water intake were recorded daily and 24 hours urine and faeces were collected.

### 4.4 Water restriction protocol

Mice were kept for 24 hours in metabolic cages (Techniplast) without access to tap water. Standard lab chow powder (Ssniff, Spezialdiäten GmbH) was mixed with water 1:1, 24 hours urine and faeces were collected and analysed. After 24 hours, the mice were put back into normal cages (T 2L (IVC), LASC, UZH).

### 4.5 Biochemical measurements

Urinary concentrations of Na^+^, K^+^, Ca^2+^ were analysed by flame photometry (EFOX 5053, Eppendorf). Urinary and plasma concentrations of Mg^2+^ were measured at the Zurich Centre for Integrative Rodent Physiology with a SYNCHRON LX^®^ System(s), UniCel^®^ DxC 800 Synchron^®^ Clinical System and Synchron^®^ System Multi Calibrator. Urinary creatinine was measured following Jaffe method. Blood gas and ion concentrations were measured in heparinized whole blood using the ABL825Flex Blood Gas Analyzer (Radiometer) immediately after blood collection from the vena cava. Plasma aldosterone levels and urinary concentration of uromodulin (UMOD) were measured with commercially available ELISA kits (Aldosterone ELISA kit by Cayman; #501090 and Mouse Uromodulin ELISA kit by Abcam; ab245726 respectively).

### 4.6 Real time quantitative polymerase chain reaction (RT‐qPCR)

RNA was isolated from kidneys and distal colon using the SV total RNA isolation kit (PROMEGA). Equal concentrations of isolated RNA (250 ng in 129Sv vs C57BL/6 or 500 ng in f/f, Δ/Δ and f/Δ) were reverse transcribed into cDNAs with the GoScript™ Reverse Transcriptase kit (PROMEGA). The generated cDNAs were further diluted with DNAse/RNAse‐free water (1:5). Primers were designed for quantification of total Slc12a1, Slc12a3, Trpm6, Trpv5, Scnn1a, Scnn1b, Scnn1g and Kcnj1 (Table S3). Quantitative PCR analysis was performed using LightCycler^®^480 (Roche) to measure expression levels of the genes of interest. Relative gene expression was calculated using the delta Ct method using β‐actin as the reference gene.

### 4.7 Western blot analysis

Kidneys were homogenized in a detergent‐free lysis buffer (DFLB) containing mannitol 200 mM, HEPES [4‐(2‐hydroxylethyl)‐1piperazineethanesulfonic acids] 80 mM, potassium hydroxide 41 mM, protease inhibitors (Complete Ultra, Roche) and phosphatase inhibitors (PhosSTOP, Roche) using 32 Magna Lyser Green Beads (Roche) in a Precellys 24 tissue homogenizer (Bertin Instruments, Montigny‐le‐Bretonneux). Beads were sedimented by centrifugation for 10 minutes (1987 rcf) and the protein containing supernatant was stored in single‐use‐aliquots at −80℃. For Western blot, 50 µg of protein was denatured in Laemmli Buffer, separated by SDS‐PAGE using 8%‐12% gels and subsequently transferred to nitrocellulose membranes. Non‐specific binding sites were blocked using 1x Odyssey Blocking Solution (Li‐COR Biosciences) before membranes were incubated with primary antibodies (Table S4) diluted in 0.2x Odyssey Blocking Solution at 4℃ for 12 hours. After washing in PBS‐0.1%Tween, the membranes were incubated with secondary antibodies (goat‐anti‐rabbit IRDye 800 or goat‐anti‐mouse IR dye 680, 1:10’000, LI‐COR Biosciences) diluted in 0.1x Casein Blocking buffer (Sigma). Detected bands were visualized with the Odyssey infrared imaging system (Li‐COR Biosciences). To ensure equal protein loading, membranes were either co‐stained with an antibody against β‐actin or total protein was stained using REVERT Total Protein Stain (Li‐COR Biosciences) prior to antibody detection. Immunoreactive bands were quantified in Fiji (ImageJ) and normalized against β‐actin signal or REVERT Total protein stain respectively.

### 4.8 Immunohistochemistry

Cryostat sections (thickness 4 µm) were blocked for non‐specific antibody binding with 10% normal goat serum and subsequently incubated with primary antibodies (Table S4) overnight at 4℃. After washing with 1X PBS, slides were incubated with secondary antibodies (Cy3 – conjugated goat‐anti‐rabbit IgG, product number 111‐165‐144, 1:1000 and FITC – conjugated goat‐anti‐mouse IgG, product number 115‐095‐068, 1:100 respectively, both from Jackson Immuno Research Laboratories) for 2 hours at room temperature. All antibodies were diluted in 1%BSA/1xPBS. Cell nuclei were stained with DAPI (4′,6‐Diamidino‐2‐phentyl‐indole, Sigma‐Aldrich, Co.) in a concentration of 0.1 µg/mL. Imaging was performed using a Leica DM6000 B fluorescence microscope (Leica Microsystems, 35578) with a Leica DFC350 FX fluorescence monochrome digital camera (Leica Microsystems, 35578). Images were processed in Fiji (ImageJ).

### 4.9 Generation of affinity purified rabbit‐anti‐mouse pT96 NKCC2 antibody

Antibody production was performed by Pineda antibody‐service (Berlin, Germany). Rabbits were immunized with a phospho‐peptide comprising the amino acids 105‐114 of the C57BL/6 mouse NKCC2 sequence (NH2‐YYLQ(p)TMDAV‐COOH). Monospecific IgG fraction was affinity‐purified against the phospho‐peptide and subsequently preabsorbed with the dephospho‐peptide resulting in a phosphoform‐specific antibody fraction. Antibody specificity of this fraction was tested by immunohistochemistry and Western blot analysis (Figure 5 and Figure S4).

### 4.10 Phosphatase treatment

Protein lysates were incubated for 1 hour at 37℃ with calf intestinal alkaline phosphatase (CIAP) (1 U/1 µg protein) to induce the hydrolysis of 5′‐phosphate groups of phosphorylated proteins, which inhibited the specific binding of the pT96 NKCC2 antibody to the proteins on nitrocellulose Western blot membrane (Figure 5B). Archived paraffin‐embedded kidneys from C57BL6 mice were used. Treatment of sections using calf intestinal alkaline phosphatase (Sigma Aldrich) was performed as described earlier with minor modifications.^56^ After blocking of free aldehyde groups, sections were rinsed five times in alkaline phosphatase buffer (100 mm Tris, 50 µm CaCl_2_, 0.1 mm MgCl_2_, 8.4 µm leupeptin, 4 mm Pefablock, pH 9.0). Later, the sections were incubated in alkaline phosphatase buffer with or without calf intestinal alkaline phosphatase (~130 units/mL) in an incubator at 35℃ for 2 hours. Immunohistochemistry and light microscopy was subsequently performed as detailed.^57^

### 4.11 Amino acids sequence alignment

The amino acids sequences for different species and mouse strains were obtained from the open data base “Ensembl Genome Browser” (www.ensembl.org, Sanger Institute).^42^ The sources of the transcript IDs are listed in Table S1. The sequences were aligned using Qiagen^©^ CLC Main Workbench 20.

### 4.12 Kidney slices

Kidney slices of C57BL/6 mice were used for ex vivo experiments as described previously.^12^ Briefly, mice were fasted overnight with free access to water to avoid any potential effects of dietary ion intake on phosphorylation levels of NKCC2 and NCC. Kidneys were removed from anaesthetized animals, cut into 280 µm thick slices using a vibrating microslicer (Vibratome, Microm; Thermo Scientific), and placed in a Ringer's solution (in mM): 98.5 NaCl, 25 NaHCO_3_, 3 KCl, 1 Na_2_HPO_4_, 2.5 CaCl_2_, 1.8 MgCl_2_ and 25 glucose for 30 minutes at 30.5℃ with constant bubbling with 95% O_2_ and 5% CO_2_. Subsequently, the Ringer solution was replaced by similar solutions but with different Cl^−^ (5 mM for low Cl^−^, 110 mM for normal Cl^−^) and K^+^ concentrations (3 mM for normal K^+^, 10 mM for high K^+^) for additional 30 minutes. Finally, slices were snap frozen in liquid nitrogen.

### 4.13 Statistics

Unpaired Student's *t* test and Welch's test in the case of unequal variances were used to compare two groups (GraphPad Prism, Version 8.0.2). For multiple comparison, one‐way or two‐way ANOVA followed by Tukey's multiple comparison post‐test was performed (GraphPad Prism, Version 8.0.2). Data are displayed as means ± SEM (in Tables and Figures). Differences were considered to be significant when *P* < .05.

## CONFLICT OF INTEREST

The group of JL receives royalties for licensed antibodies against NCC and phosphorylated Nedd4‐2 from Milipore and Abcam respectively. Both antibodies were not used in the current study. Moreover, JL received 2019 an honorarium from VIFOR for an oral presentation at a symposium organized by VIFOR.

## Supporting information

Supplementary Material

## Data Availability

The data that support the findings of this study are available from the corresponding author upon reasonable request.
