# Supplementary material for: A five amino acids deletion in NKCC2 of C57BL/6 mice affects analysis of NKCC2 phosphorylation but does not impact kidney function
Source: Acta Physiol (Oxf). 2021 Jun 26;233(1):e13705. doi: 10.1111/apha.13705 (PMC8384713; doi:10.1111/apha.13705)
Supplement: Supplementary file 1 — Supplementary Material [file APHA-233-e13705-s001.docx]

**Supplementary Material - Table of content:**

- Supplementary Material and Methods
- Supplementary References
- Supplementary Table 1: Transcript IDs of protein sequences for *Slc12a1* and *Slc12a3* of different species
- Supplementary Table 2: Primers used for cDNA sequencing of exon 2 of C57BL/6 NKCC2
- Supplementary Table 3: Primers used for genotyping and qPCR
- Supplementary Table 4: Primary antibodies used in experiments and their dilutions for Western blot and immunofluorescence
- Supplementary Figure 1: Nucleotide sequence alignment of Slc12a1 exon 2 of 129Sv and C57BL/6 mice.
- Supplementary Figure 2: Several phosphoform-specific NKCC2 antibodies cross-react with phosphoform-specific NCC
- Supplementary Figure 3: Impact of tissue processing on the immunodetection of total NKCC2 and phosphorylated NKCC2
- Supplementary Figure 4: Cross-reactivity of pNCC antibodies with pNKCC2 in cryosections of kidneys from 129Sv but not from C57BL/6 mice
- Supplementary Figure 5: Daily food intake, fecal output and fecal Mg^2+^ and Ca^2+^ loss
- Supplementary Figure 6: TRPM6 and TRPM7 mRNA expression in colon
- Supplementary Figure 7: Bone mineral density in 129Sv and C57BL/6 mice
- Supplementary Figure 8: Specificity of pT96 NKCC2 antibody
- Supplementary Figure 9: Impact of low chloride stimulation and loaded amount of protein on the immunodetection of total NKCC2 and phosphorylated NKCC2
- Supplementary Figure 10: Full gels of Western blots Fig. 1e and 1f
- Supplementary Figure 11: Full gels of Western blots Fig. 2b
- Supplementary Figure 12: Full gels of Western blots Fig. 4b
- Supplementary Figure 13: Full gels of Western blots Fig. 5a and 5b
- Supplementary Figure 14: Full gels of Western blots Fig. 6b
- Supplementary Figure 15: Full gels of Western blots Fig. 7a and 7c
- Supplementary Figure 16: Full gels of Western blots Supplementary Fig. 2
- Supplementary Figure 17: Full gels of Western blots Supplementary Fig. 3
- Supplementary Figure 18: Full gels of Western blots Supplementary Fig. 9

**Supplementary Material and Methods**

**Nucleotide sequencing of DNA from C57BL/6 mice**

C57BL/6 mice were obtained from a commercial breeder in the US (The Jackson Laboratory, Bar Harbor, ME) and a commercial breeder in Europe (Janvier Labs, Le Genest-Saint-Isle, France). For US C57BL/6J mice, genomic DNA extracts were prepared from tail snips by heating overnight at 55°C in 300 μL of digestion solution containing 5 mM EDTA, 200 mM NaCl, 100 mM Tris (pH 8.0), 0.2% SDS, and 0.4 mg/mL proteinase K, followed by ethanol precipitation. DNA from exon 2 of mouse NKCC2 was amplified using the primers described in Suppl. Table 2 and sequenced by Genewiz Inc (Seattle, US) using the primer 5’-CCCCACATTATGAAGAGACCTC-3’. For Europe C57BL/6J-Rj and 129S2/SvPasOrlRj mice, genomic DNA was extracted from a kidney slice by heating for 20 minutes at 95 °C in 50 mM NaOH followed by vigorous vortexing. The genomic region spanning exon 2 of mouse NKCC2 was amplified using the primers provided in Suppl. Table 2 and sequenced by Microsynth AG (Balgach, Switzerland) using the same primers as for PCR.

**Different processing of kidney samples**

Kidneys were divided into four pieces. Two pieces were directly frozen and stored at -80° C for later processing. The two other pieces were directly processed. One piece was lysed with our standard detergent free lysis buffer (DFLB) containing mannitol 200 mM, HEPES [4-(2-hydroxylethyl)-1piperazineethanesulfonic acid] 80 mM, potassium hydroxide 41 mM), while the second piece was lysed with the detergent-containing RIPA (Pierce™ RIPA Buffer, Thermo Scientific, Waltham, MA) buffer. Both buffers were supplemented with protease inhibitors (Complete Ultra, Roche) and phosphatase inhibitors (PhosSTOP, Roche).

**Measurement of fecal magnesium and calcium content**

Mice were placed in metabolic cages and feces were collected for 48h. Frozen samples of the feces were shipped to ALS Scandinavia AB (Luleå, Sweden) andmagnesium and calcium measurements were performed by using inductively coupled plasma sector field mass spectrometry (ICP-SFMS).

**Bumetanide and hydrochlorothiazide test**

Mice were adapted to metabolic cages for three days. Then, mice were injected i.p. with 10 μl /g BW of vehicle and urine was collected over the next 4h. The following day, mice received either a single i.p. injection (10 μl /g BW) of bumetanide (Sigma-Aldrich, B3021-1; 40mg/kg BW dissolved in 40% PEG300 and 60% NaCl 0.9%) or of hydrochlorothiazide (Sigma-Aldrich, H4759-5G; 40mg/kg BW dissolved in 40% DMSO and 60% NaCl 0.9%). Urine was again collected over the next 4h.

**Micro-Computed Tomography (micro-CT)**

Femur, tibia and fibula were harvested *in toto* from dead mice and were then frozen and stored at -20°C. Bone microstructure and bone mineral density analyses were performed by using high-resolution micro-Computed Tomography (micro-CT, Quantum Fx, Perkin Elmer, Hopkinton, MA, USA) by Dr. Petra Seebeck (Zurich Integrative Rodent Physiology (ZIRP), UZH, Zurich, CH) as described previously.^1^

**MDCK cell lines**

The MDCKI-hNKCC2A and -mNKCC2 C57BL/6 cell lines were generated by introducing a FLAG-tag (GACTACAAGGACGATGATGACAAG; amino acids DYKDDDDK) into the NH2 terminus of either a human NKCC2 isoform A (hNKCC2A) or mouse NKCC2 C57BL/6 (mNKCC2 C57BL/6) cDNA by standard site directed mutagenesis approaches and subcloned into a pcDNA5/FRT/TO/TOPO vector (Invitrogen). Plasmids were co-transfected using Lipofectamine 2000 (Thermo Scientific) with pOG44 (encoding flp recombinase) into tetracycline inducible MDCK type I cell lines containing a single FRT site in its genome.^2^ Positive clones were selected using 300 μg/ml Hygromycin B and stable MDCKI-hNKCC2A and MDCKI-mNKCC2 C57BL/6 cell lines were maintained in DMEM high glucose with 10% DBS, 150 μg/ml Hygromycin B, and 5 μg/ml Blasticidin HCl (all Thermo Scientific). The tetracycline inducible MCDKI-hNCC cell line was generated and cultured as previously described.^3^ Protein samples were isolated from cells grown on plastic induced with or without 10µg/ml tetracyline HCl and 5 mM valproic acid for 24 hours. Cells were washed in DMEM high glucose and stimulated with either hypotonic low chloride buffer (67.5 mM sodium gluconate, 2.5 mM potassium gluconate, 0.5 mM CaCl_2_, 0.5 mM MgCl_2_, 1 mM Na_2_HPO_4_, 1 mM Na_2_SO_4_, 7.5 mM sodium HEPES, pH 7.4) or 25 µM forskolin for 20 min at 37°C. Samples were separated by SDS-PAGE using 4–15% gradient polyacrylamide gels (Criterion TGX Precast Protein Gels, BioRad). Immunoblots were developed using SuperSignal West Femto chemiluminescent substrate (Thermo Scientific) or Amersham ECL Western Blotting Detection Reagent (GE Healthcare) detection.

**Immunoprecipitation from cell lines**

Cells were grown in DMEM high Glucose with 10% DBS on filter plates until confluent. For MDCKI-hNCC, plates were coated with basement membrane extract (BME) (Cultrex® Basement Membrane Extract, PathClear, R&D Systems). Cells were induced with or without 10µg/ml tetracyline HCl and 5 mM valproic acid for 16–24 hrs prior to experiment. Cells were washed twice in isotonic buffer (135 mM NaCl, 5 mM KCl, 1 mM CaCl_2_, 1 mM MgCl_2_, 1 mM Na_2_HPO_4_, 1 mM Na_2_SO_4_, 15 mM sodium HEPES, pH 7.4) before stimulation with hypotonic low chloride buffer for 20 min at 37 °C. Cells were subsequently biotinylated at the apical plasma membrane, the biotinylated fraction was isolated and subjected to IP using 2 µg of FLAG antibody (Sigma, F7425) and 20 µl protein A-agarose or 40 µl Anti-FLAG M2 affinity gel (Sigma, A2220) as previously described.^4^ Samples were separated by SDS-PAGE as described above. Antibodies used were a rabbit polyclonal antibody against total NKCC2 (#1495;^5^), the newly generated pT96 NKCC2 (as described), a mouse monoclonal antibody against total NCC (kind gift from Henrik Dimke, University of Southern Denmark, Denmark) and proteasome 20S (ab3325, Abcam).

**Supplementary Table 1:**

| Species | Gene of interest | Transcript ID source |
| --- | --- | --- |
| *Homo sapiens* | *Slc12a1* | [ENST00000647546.1](https://www.ensembl.org/Homo_sapiens/Transcript/Sequence_Protein?db=core;g=ENSG00000074803;r=15:48178438-48304078;t=ENST00000647546) |
| *Homo sapiens* | *Slc12a3* | [ENST00000438926.6](https://www.ensembl.org/Homo_sapiens/Transcript/Sequence_Protein?db=core;g=ENSG00000070915;r=16:56865207-56915850;t=ENST00000438926) |
| *Gallus gallus domesticus* | *Slc12a1* | [ENSGALT00000007915.6](https://www.ensembl.org/Gallus_gallus/Transcript/Summary?db=core;g=ENSGALG00000004945;r=10:10537715-10583316;t=ENSGALT00000007915) |
| *Gallus gallus domesticus* | *Slc12a3* | [XP_414059.4](https://www.ensembl.org/Gallus_gallus/Transcript/Summary?db=core;g=ENSGALG00000002957;r=11:2036749-2043464;t=ENSGALT00000004672) |
| *Rattus norvegicus* | *Slc12a1* | [ENSDART00000165073.2](https://www.ensembl.org/Danio_rerio/Transcript/Sequence_Protein?db=core;g=ENSDARG00000098096;r=18:5273953-5300028;t=ENSDART00000165073) |
| *Rattus norvegicus* | *Slc12a3* | [ENSDART00000186977.1](https://www.ensembl.org/Danio_rerio/Transcript/Sequence_Protein?db=core;g=ENSDARG00000013855;r=18:17583479-17598755;t=ENSDART00000186977) |
| *Danio rerio* | *Slc12a1* | XP_021323409.1 |
| *Danio rerio* | *Slc12a3* | [ENSDART00000186977.1](https://www.ensembl.org/Danio_rerio/Transcript/Sequence_Protein?db=core;g=ENSDARG00000013855;r=18:17583479-17598755;t=ENSDART00000186977) |
| *Xenopus tropicalis* | *Slc12a1* | XP_012814933.2 |
| *Xenopus tropicalis* | *Slc12a3* | XP_002937217.2 |
| Mice | Gene of interest | Transcript ID source |
| 129S1SvImJ | *Slc12a1* | [MGP_129S1SvImJ_T0057357.1](https://www.ensembl.org/Mus_musculus_129S1_SvImJ/Transcript/Sequence_Protein?db=core;g=MGP_129S1SvImJ_G0026426;r=2:128316574-128400680;t=MGP_129S1SvImJ_T0057357) |
| 129S1SvImJ | *Slc12a3* | [MGP_129S1SvImJ_T0090010.1](https://www.ensembl.org/Mus_musculus_129S1_SvImJ/Transcript/Summary?db=core;g=MGP_129S1SvImJ_G0033959;r=8:95744294-95781353;t=MGP_129S1SvImJ_T0090010) |
| C57BL/6 | *Slc12a1* | [MGP_C57BL6NJ_T0057800.1](http://www.ensembl.org/Mus_musculus_C57BL_6NJ/Transcript/Summary?db=core;g=MGP_C57BL6NJ_G0026847;r=2:132427097-132508397;t=MGP_C57BL6NJ_T0057800) |
| C57BL/6 | *Slc12a3* | [MGP_C57BL6NJ_T0090549.1](https://www.ensembl.org/Mus_musculus_C57BL_6NJ/Transcript/Summary?db=core;g=MGP_C57BL6NJ_G0034455;r=8:99239270-99276289;t=MGP_C57BL6NJ_T0090549) |

**Supplementary Table 1: List of transcript IDs used for amino acids sequence alignment of different species for *Slc12a1* and *Slc12a*3**

**Supplementary Table 2:**

| Target Gene | Forward primer | Reverse primer |
| --- | --- | --- |
| *Slc12a1* exon 2 of C57BL/6J (USA) | 5’-GGTCCATGTTATAAAG AGGGCCATGG-3’ | 3’-GGAACAGCAGGAAGGATGC TTAC-5’ |
| *Slc12a1* exon 2 of C57BL/6J-Rj (Europe) and 129S2/SvPasOrlRj (Europe) | 5'-GTCGCTTTCAGGTCCATG TT-3' | 5'-AACAGCACGGAAGGATGC-3' |

**Supplementary Table 2: Primer sequences for nucleotide sequencing of exon 2 of *Slc12a1* in C57BL/6J, C57BL/6J-Rj and 129S2/SvPasOrlRj mice**

**Supplementary Table 3:**

| Purpose | Target | Forward primer (5’-3’) | Reverse primer (3’-5’) |
| --- | --- | --- | --- |
| qPCR | *Slc12a1* (NKCC2) | GGAATTGGTCTGGGCGTCA | ATTGACCCACCGAACTCAGG |
| qPCR | *Slc12a3* (NCC) | TGACCTGCATTCATTCCTCA | GAAGCGAACAGGTTCTCCAG |
| qPCR | *Trpm6* | CCTTGGGGAGTCATTGAGAAC | CAGTCCCATCATCACACAGG |
| qPCR | *Trpv5* | CCACAGTGATGCTGGAGAGG | GGATTCTGCTCCTGGTGG TG |
| qPCR | *Scnn1a* (α-ENaC) | ACCCCGTGAGTCTCAACATC | CCTGGCGAGTGTAGGAAGAG |
| qPCR | *Scnn1b* (β-ENaC) | TTCAACTGGGGCATGACAG | CCGATGTCCAGGATCAATT |
| qPCR | *Scnn1g* (γ-ENaC) | AGTTCAGAAAGAACTCTGCAGGC | GTGTTCAGGCAGTACCAATGC |
| qPCR | *Kcnj1* (ROMK) | CTTGGGGACCAAGAAAATGA | TCCATCTTTGGAGACCAACC |
| Genotyping | *Slc12a1*  (NKCC2) | CGCCTACGACTCTCACAC | GCGGTAGTATTCAATCTTGG |

**Supplementary Table 3: List of primer sequences for qPCR of genes of interest as well as for the genotyping of the f/f vs Δ/Δ mice**

**Supplementary Table 4:**

| Antibody | Host | Dilution WB | Dilution IHC | Source |
| --- | --- | --- | --- | --- |
| Anti-total NCC | Rabbit | 1:10,000 | 1:2000 | ^6^ |
| Anti-total NCC | Mouse | 1:1000 |  | Unpublished, Henrik Dimke |
| Anti-phospho-Thr53 NCC | Rabbit | 1:2000 |  | ^6^ |
| Anti-phospho-Thr58 NCC | Rabbit | 1:5000 |  | ^6^ |
| Anti-total-NKCC2 | Rabbit | 1:5000 | 1:20,000 | ^7^ |
| Anti-total-NKCC2 | Rabbit | 1:250 |  | ^5^ |
| Anti-mouse-phospho-Thr96/Thr101-NKCC2 | Rabbit | 1:5000 | 1:4000 and  1:10,000 | ^8^ |
| Anti-human-phospho-Thr212/Thr217-NKCC1 “R5” | Rabbit | 1:5000 |  | ^9^ |
| Anti-rat-phospho-Thr96/Thr101-NKCC2 | Rabbit | 1:5000 |  | ^10^ |
| Anti-mouse-phospho-Thr96-NKCC2 | Rabbit | 1:500 | 1:6000 | This study |
| Anti-α-ENaC | Rabbit | 1:5000 |  | ^6^ |
| Anti-β-ENaC | Rabbit | 1:20,000 |  | ^7^ |
| Anti-γ-ENaC | Rabbit | 1:20,000 |  | ^7^ |
| Anti-ROMK | Rabbit | 1:1000 |  | ^11^ |
| Anti-totalSPAK | Rabbit | 1:250 |  | Milipore, 07-2271 |
| Anti-TRPM6 | Rabbit | 1:2000 |  | ^12^ |
| Anti-TRPV5 | Rabbit | 1:1000 |  | ^13^ |
| Proteasome 20S | Rabbit | 1:10,000 |  | Abcam,Ab3325 |
| Flag antibody | Rabbit | 1:750 |  | Sigma, F7425 |

**Supplementary Table 4: List of primary antibodies used for experimental analyses**

**Supplementary Figure 1:**

| 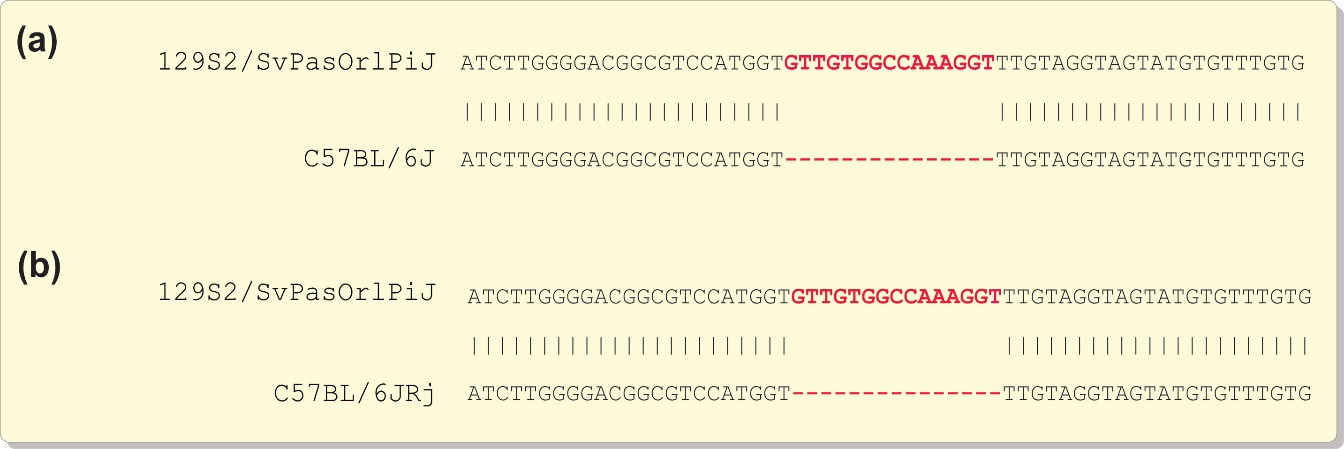 |
| --- |

**Supplementary Figure 1: Nucleotide sequence alignment of *Slc12a1* exon 2 of 129Sv and C57BL/6 mice**

**(a)** C57BL/6J mice from The Jackson Laboratory (USA) and **(b)** C57BL/6JRj mice from Janvier Labs (France) present with a 15-base deletion in exon 2, corresponding to the 5 amino acids deletion. The deletion of 15 bases is marked in red.

**Supplementary Figure 2:**

| 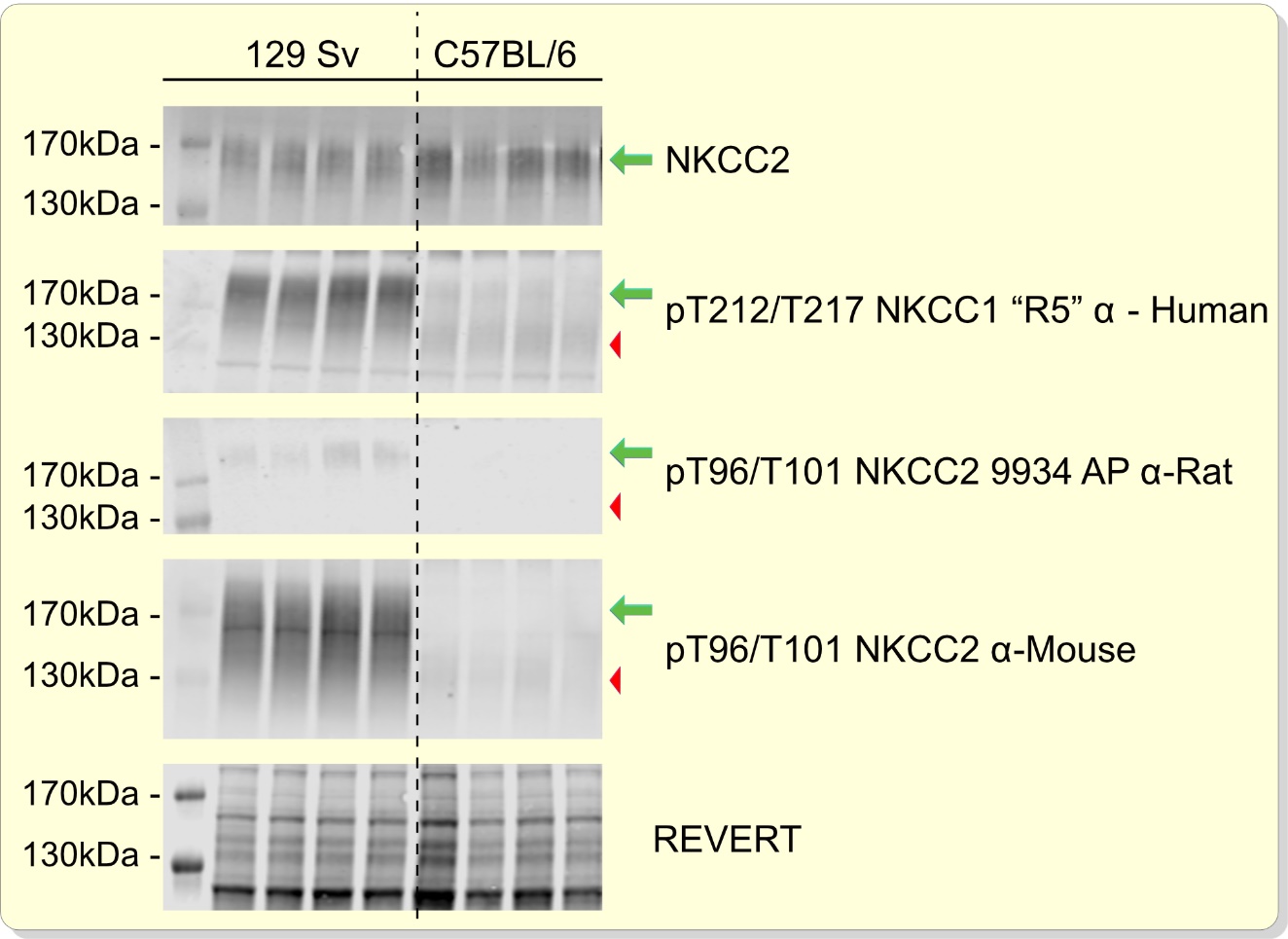 |  |
| --- | --- |

**Supplementary Figure 2: Several phosphoform specific NKCC2 antibodies cross-react with phosphoform-specific NCC**

Total NKCC2 and pNKCC2 probed in whole kidney homogenates from 129Sv and C57BL/6 mice by two previously published phosphoform-specific antibodies, pT212/T217 NKCC1 R5^9^ and pT96/T101 NKCC2 9934 AP^10^, targeting the same regulatory site but have been raised against NKCC2 of different species. Similar to our pT96/T101 NKCC2 antibody, these antibodies did not detect pNKCC2 in C57BL/6 mice (Supplementary Fig.1). n = 5 per group.

**Supplementary Figure 3:**

| 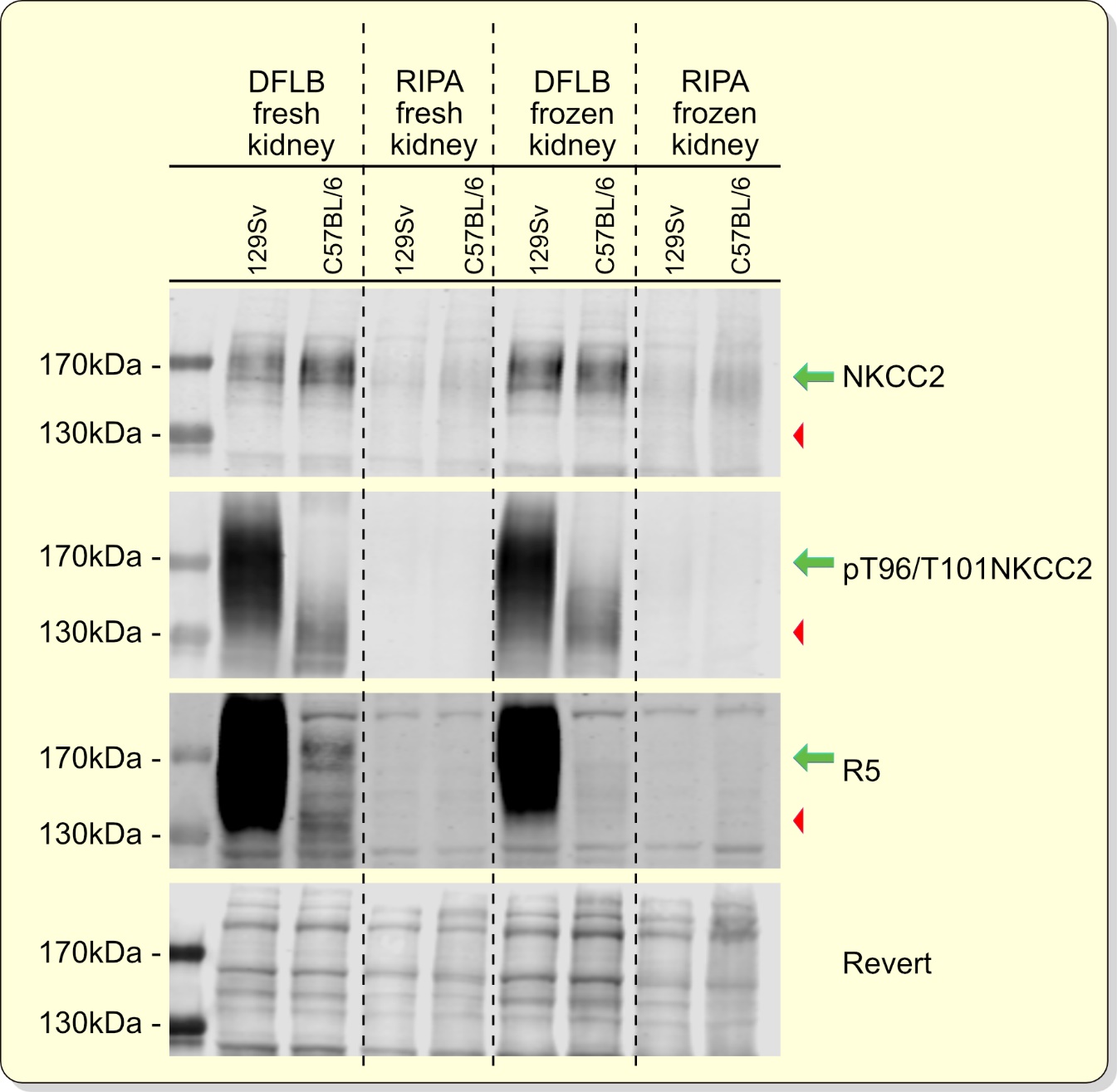 |
| --- |

**Supplementary Figure 3: Impact of tissue processing on the immunodetection of total NKCC2 and phosphorylated NKCC2**

Kidneys from 129Sv and C57BL/6 mice were harvested and processed either directly (fresh kidneys) or snap-frozen and stored at -80°C until use (frozen kidneys). Fresh and frozen kidneys were lysed either with our standard detergent-free lysis buffer (DFLB) or with a commercially available detergent-containing RIPA lysis buffer. Lysed protein samples were separated by SDS-PAGE and probed with the indicated antibodies. Revert total protein detection served as a loading control. Green arrows point to NKCC2. Red arrow-heads point to NCC.

**Supplementary Figure 4:**

| 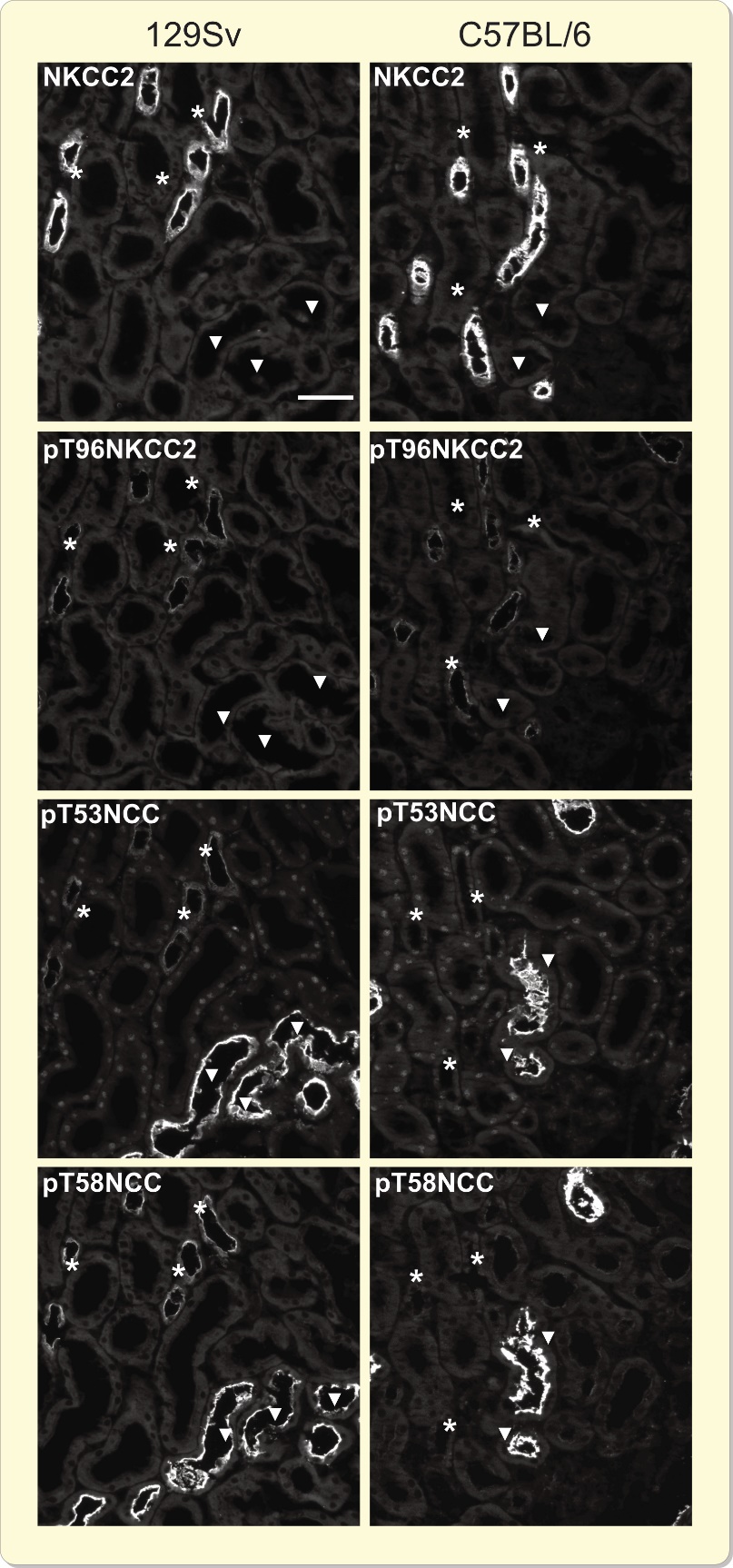 |
| --- |

**Supplementary Figure 4: Cross-reactivity of pNCC antibodies with pNKCC2 in cryosections of kidneys from 129Sv but not from C57BL/6 mice**

Cryosections were incubated with antibodies against NKCC2, pT96NKCC2, pT53NCC, and pT58NCC as indicated. Bar = 50µm, * = TAL, ▼ = DCT.

**Supplementary Figure 5:**

| **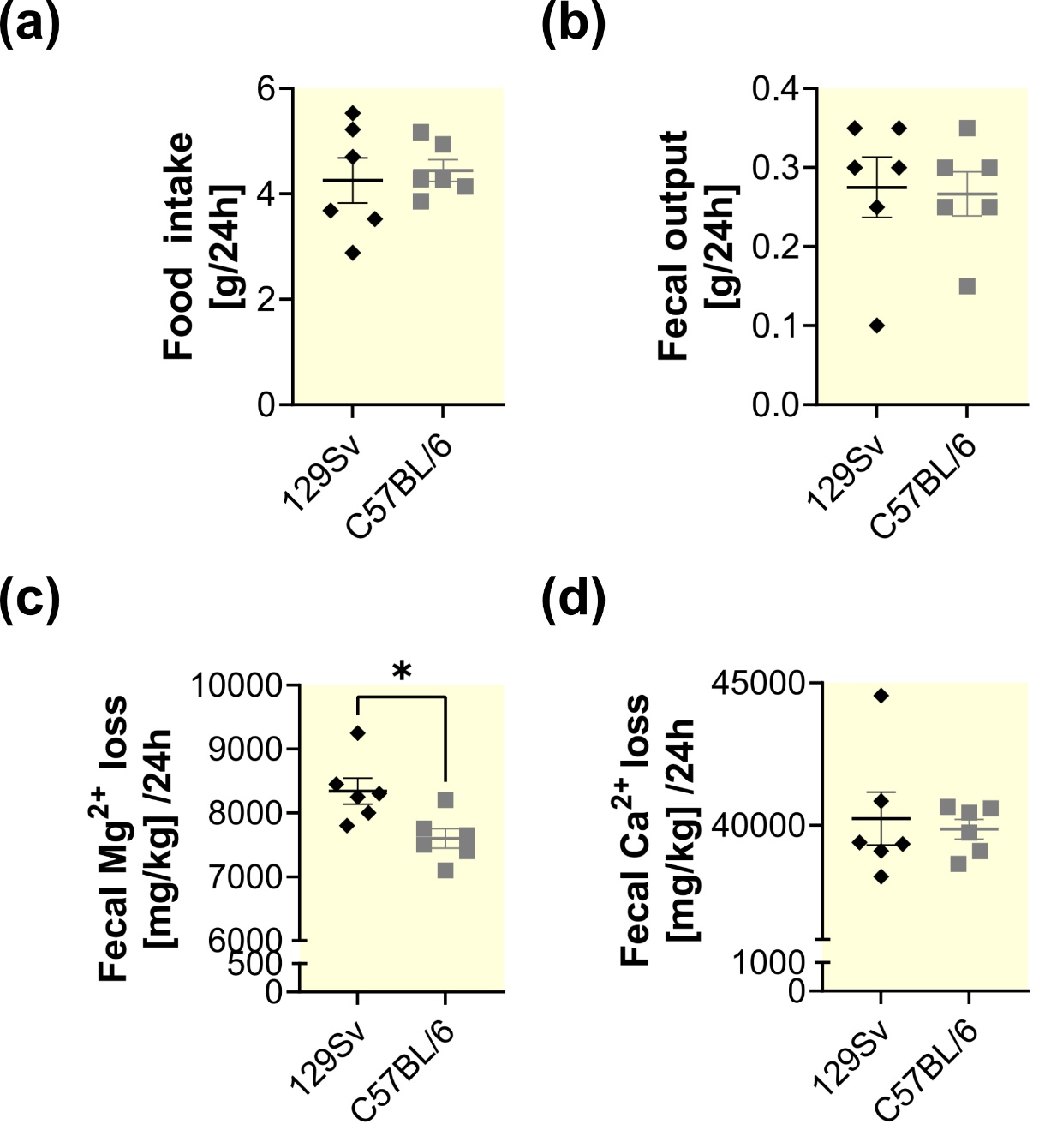** |
| --- |

**Supplementary Figure 5: Daily food intake, fecal output and fecal Mg^2+^ and Ca^2+^ loss**

**(a)** Daily (24h) food intake and **(b)** fecal output in 129Sv and C57BL/6 mice. **(c)** Daily fecal Mg^2+^ and **(d)** fecal Ca^2+^ loss of 129Sv and C57BL/6 mice. Food intake and fecal output were monitored in metabolic cages over 48h but for sake of clarity are presented here as the calculated daily intake and output; n = 5 mice per group, shown are means ± SEM.

**Supplementary Figure 6:**

| 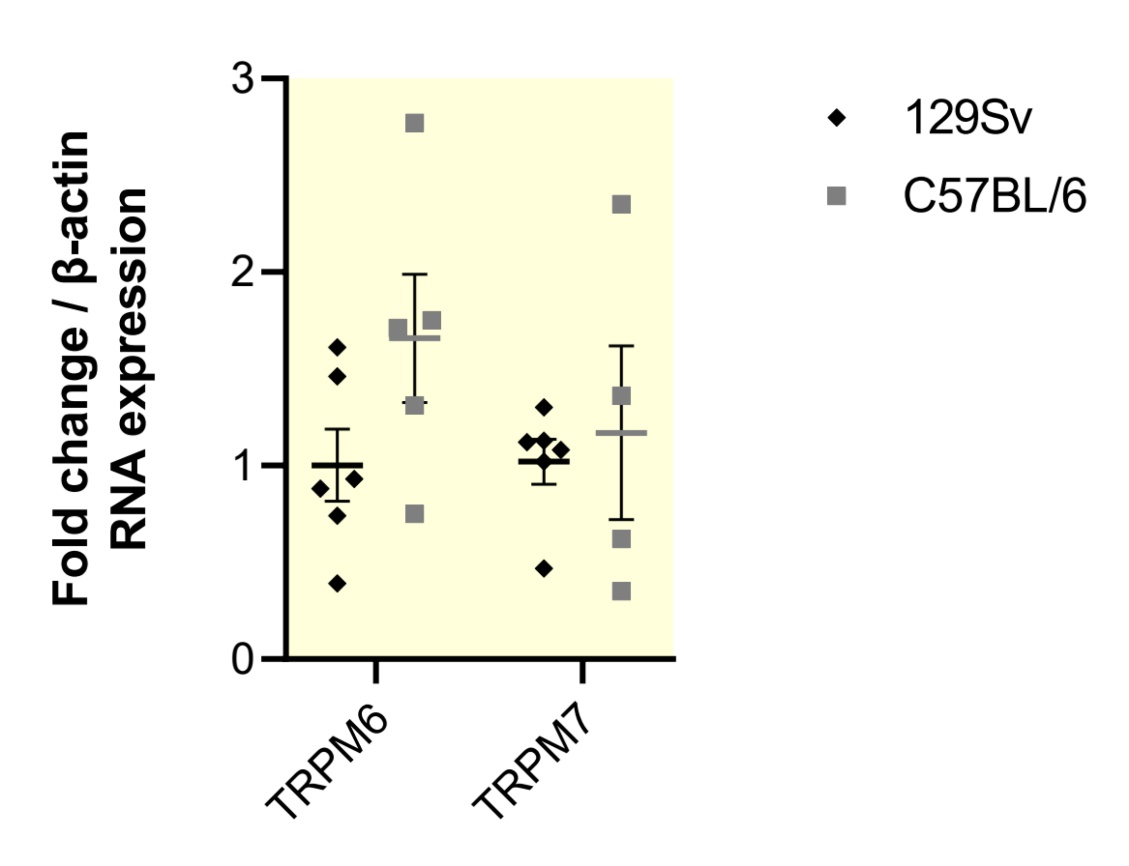 |
| --- |

**Supplementary Figure 6: TRPM6 and TRPM7 mRNA expression in colon**

Distal colons were isolated from 129Sv and C57BL/6 mice and were then analyzed by qRT-PCR for mRNA expression of the magnesium channels TRPM6 and TRPM7. Data are expressed as a fold change from the average expression levels of TRPM6 and TRPM7 mRNAs in 129Sv mice; n = 4-6 mice per group, shown are means ± SEM.

**Supplementary Figure 7:**

| 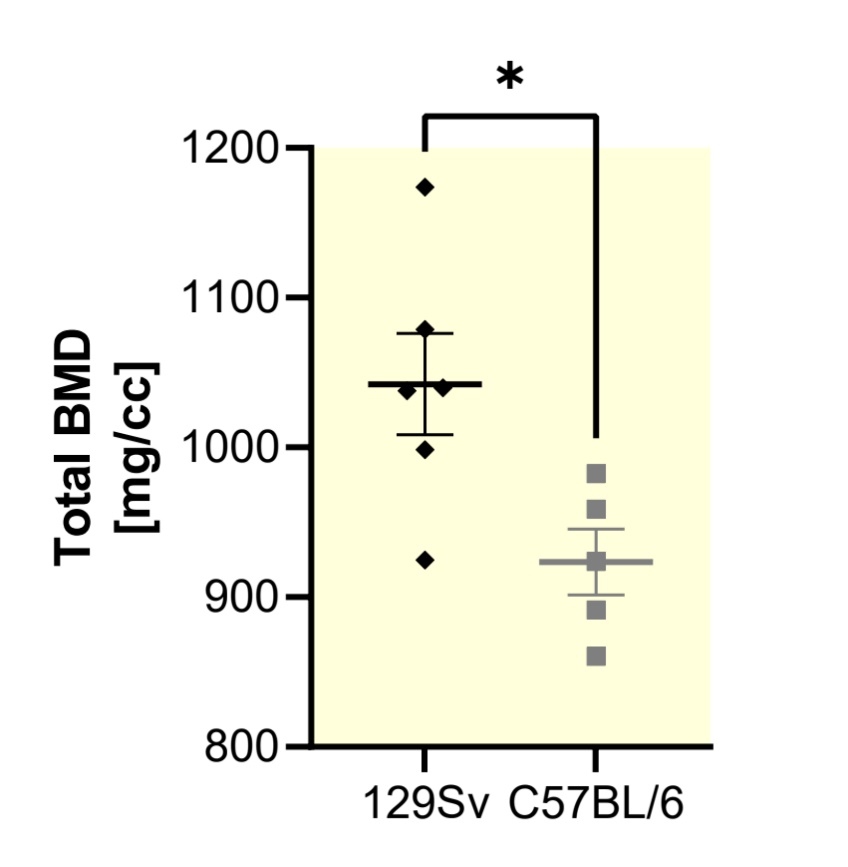 |
| --- |

**Supplementary Figure 7: Bone mineral density in 129Sv and C57BL/6 mice**

Bones from the hind limbs of 129Sv and C57BL/6 were harvested and analyzed by micro-CT as described in the supplementary methods. The total bone mineral density (BMD) is higher in 129Sv mice than in C57BL/6 mice. n = 5-6 mice per group, shown are means ± SEM.

**Supplementary Figure 8:**

| 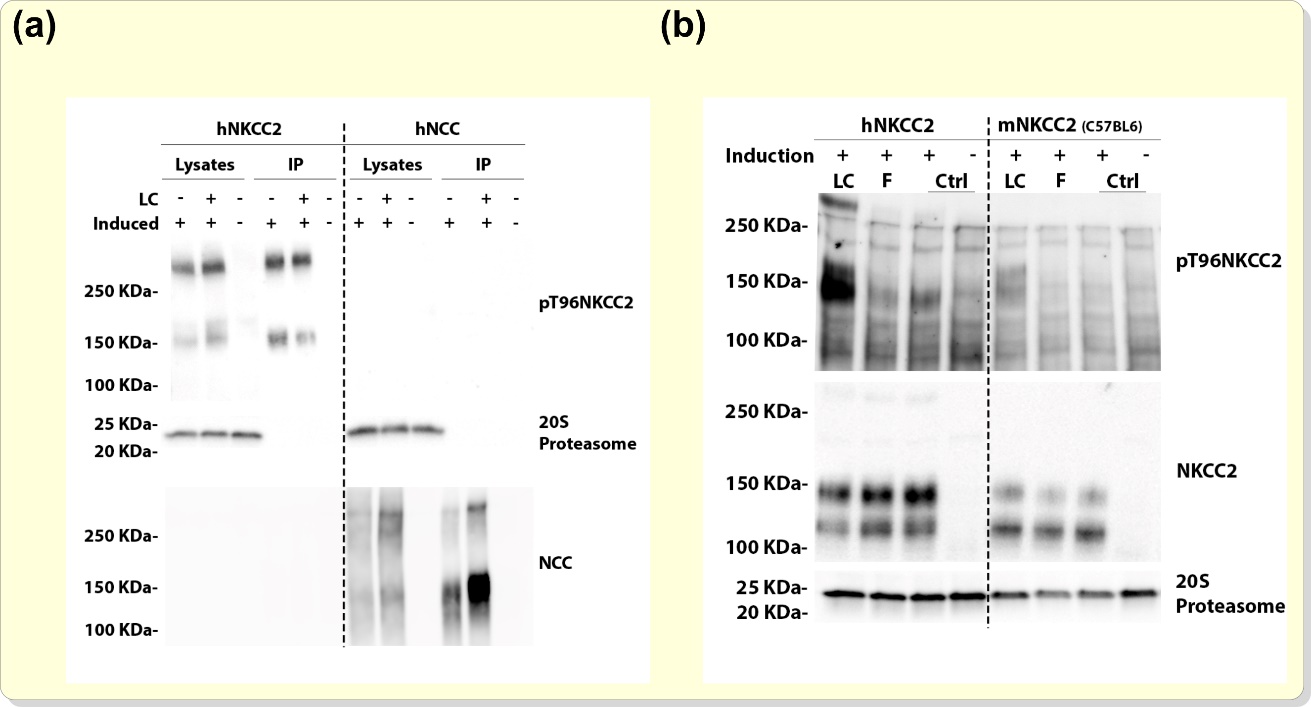 |
| --- |

**Supplementary Figure 8: Specificity of pT96 NKCC2 antibody**

**(a)** Immunoblots of IP and total lysate samples from MDCKI-FLAG-hNKCC2 (hNKCC2) and -hNCC (hNCC) cells. Cells were induced with or without tetracycline and valproic acid for 16-24H at 37°C, and subsequently stimulated with hypotonic low chloride solution (LC). Cells were subjected to biotinylation at the apical plasma membrane followed by IP using Flag antibodies. The pT96 NKCC2 antibody recognizes human NKCC2 but not human NCC. **(b)** Immunoblots of protein samples isolated from MDCKI cell lines expressing human NKCC2 (hNKCC2) or mouse NKCC2 (mNKCC2(C57BL/6)). Cells were induced with or without tetracycline and valproic acid for 24 h at 37°C, and subsequently stimulated with either a hypotonic low chloride solution (LC) or 25 µM forskolin (F) for 20 min at 37°C. The pT96 NKCC2 antibody recognizes both human and mouse NKCC2 with a clear increase in signal when stimulated with hypotonic low chloride.

**Supplementary Figure 9:**

| 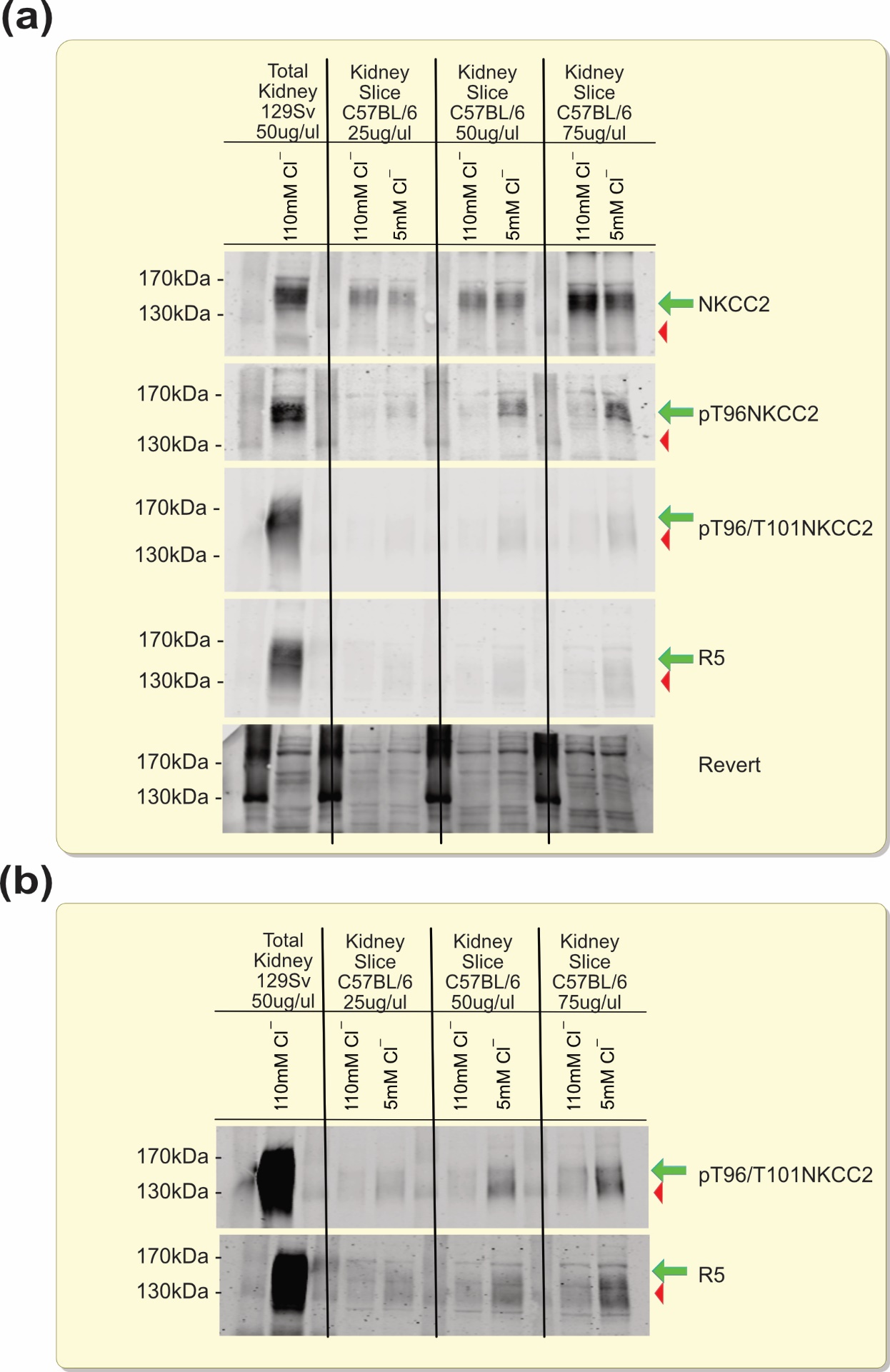 |
| --- |

**Supplementary Figure 9: Impact of low chloride stimulation and loaded amount of protein on the immunodetection of total NKCC2 and phosphorylated NKCC2**

**(a, b)** Kidney slices from C57BL/6 mice were incubated *ex vivo* either in a buffer with normal chloride concentrations (110 mM Cl^-^) or in a buffer with low chloride concentrations (5 mM Cl^-^). The kidney slices were then lysed with our standard detergent-free lysis buffer and loaded for SDS-PAGE together with a lysate from a kidney of a 129Sv mouse. The amounts of proteins loaded per lane and the antibodies used for detection of total and phosphorylated NKCC2 are indicated on the top and on the side of blots, respectively. Detection of total proteins with Revert is shown as a visual control for protein loading. Green arrows point to NKCC2 and red arrow-heads point to NCC. Panel **(a)** shows blots imaged with an Odyssey infrared imaging system (Li-COR) at standard conditions. Panel **(b)** shows the same blots for the pT96T101 NKCC2 and the R5 antibody as in **(a)**, but now imaged with enhanced settings.

**Supplementary Figure 10:**

| **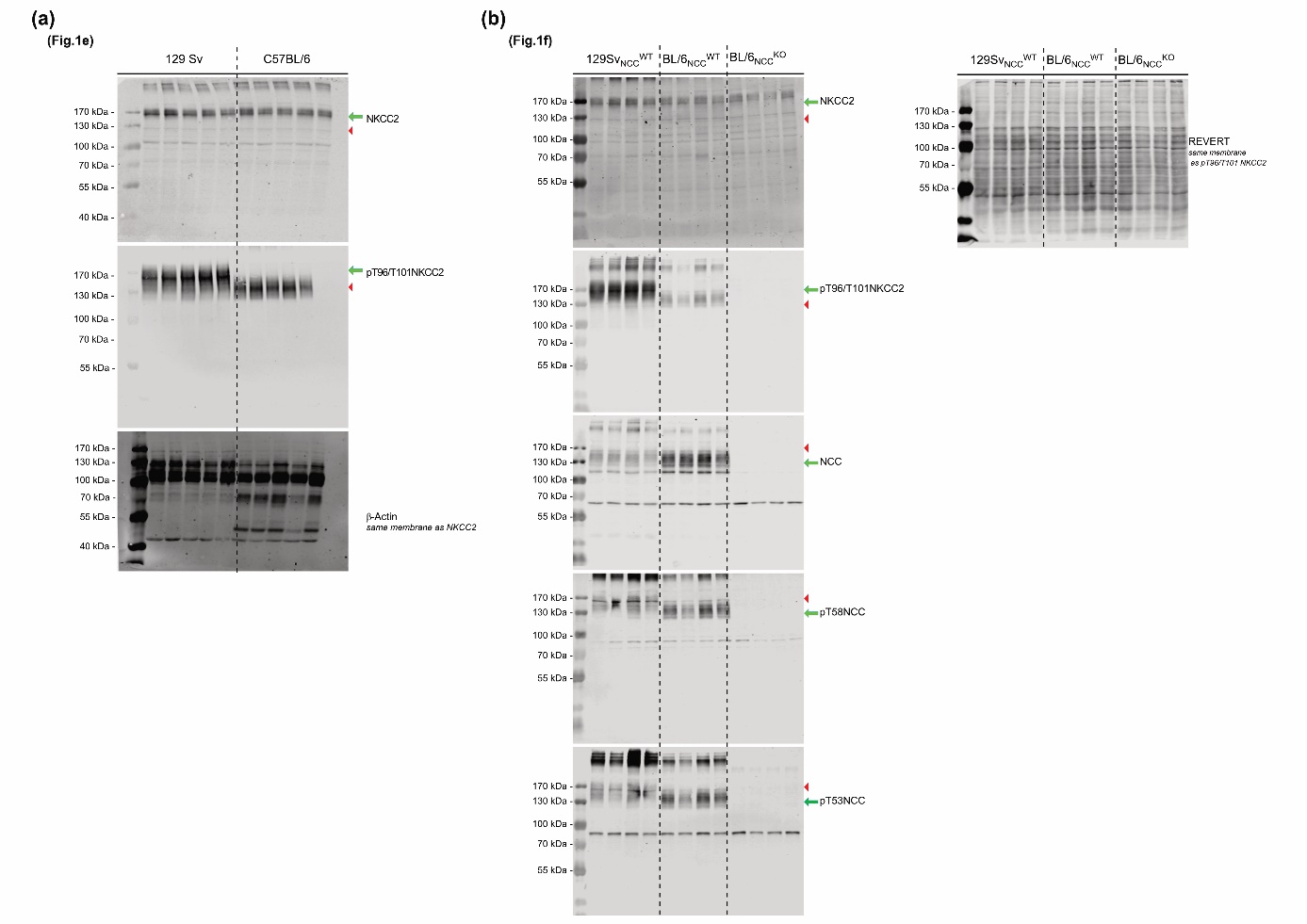** |
| --- |

**Supplementary Figure 10: Full gels of Western blots Fig. 1e and 1f**

**Supplementary Figure 11:**

| 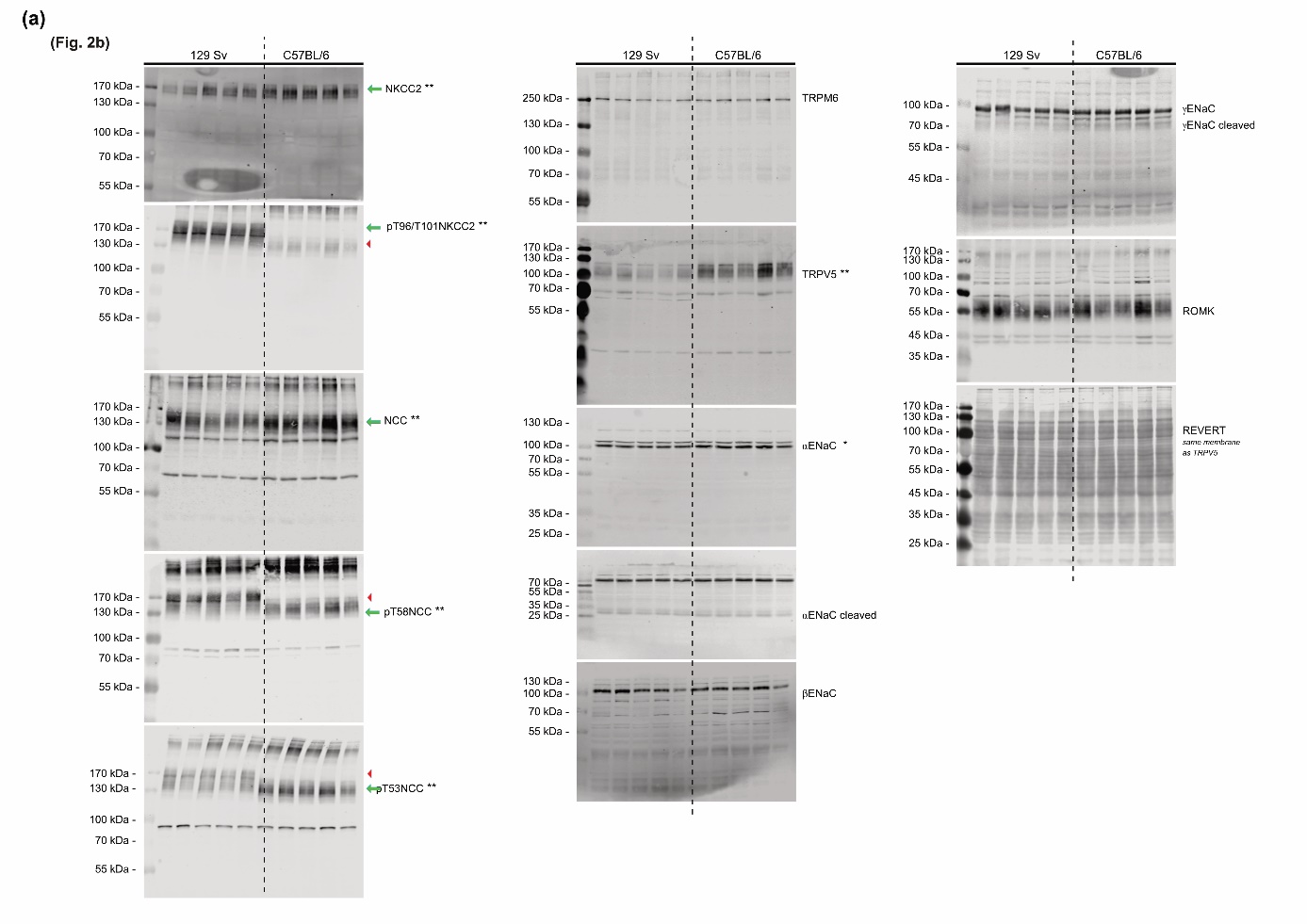 |
| --- |

**Supplementary Figure 11: Full gels of Western blots Fig. 2b**

**Supplementary Figure 12:**

| **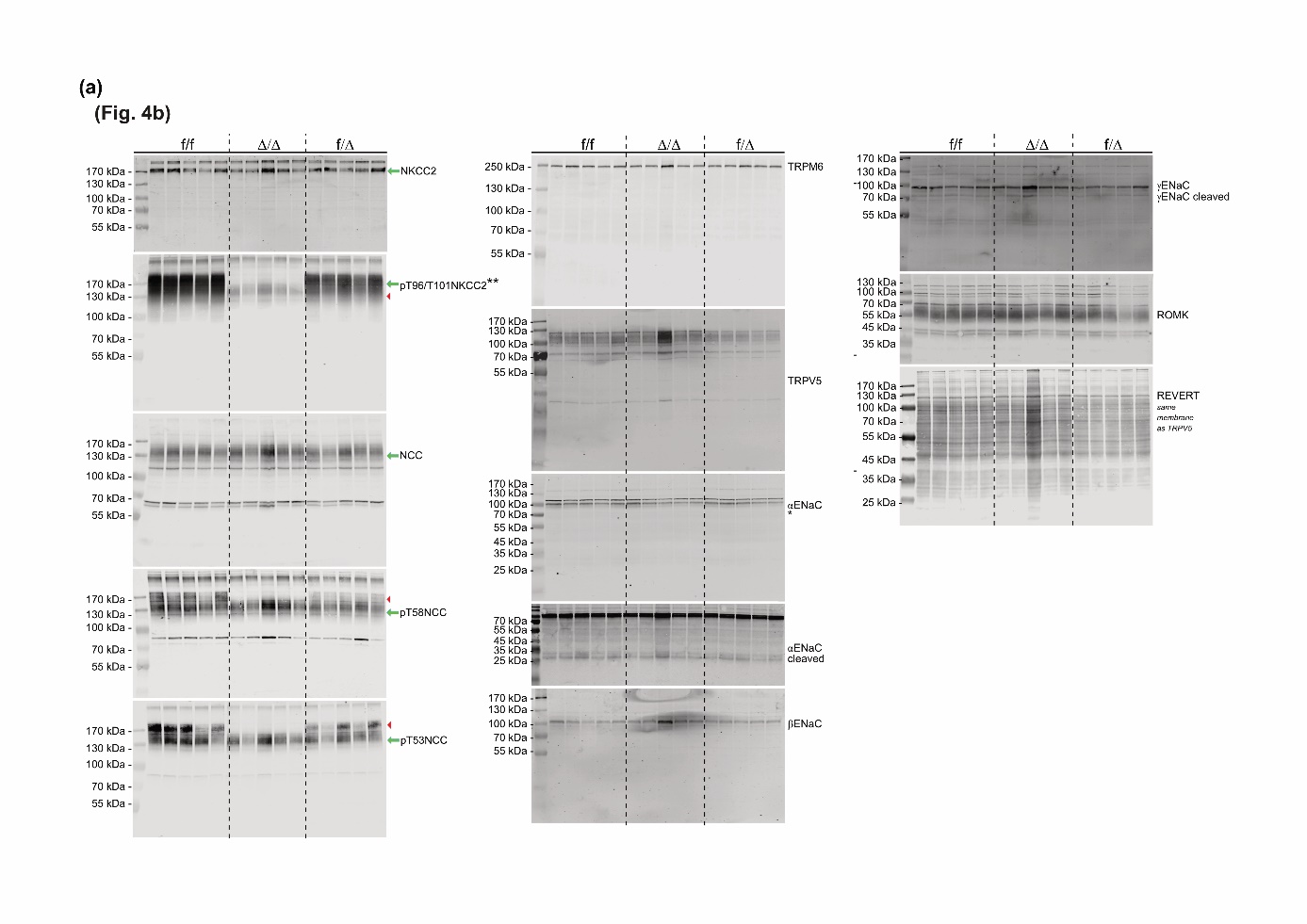** |
| --- |

**Supplementary Figure 12: Full gels of Western blots Suppl. Fig. 4b**

**Supplementary Figure 13:**

| **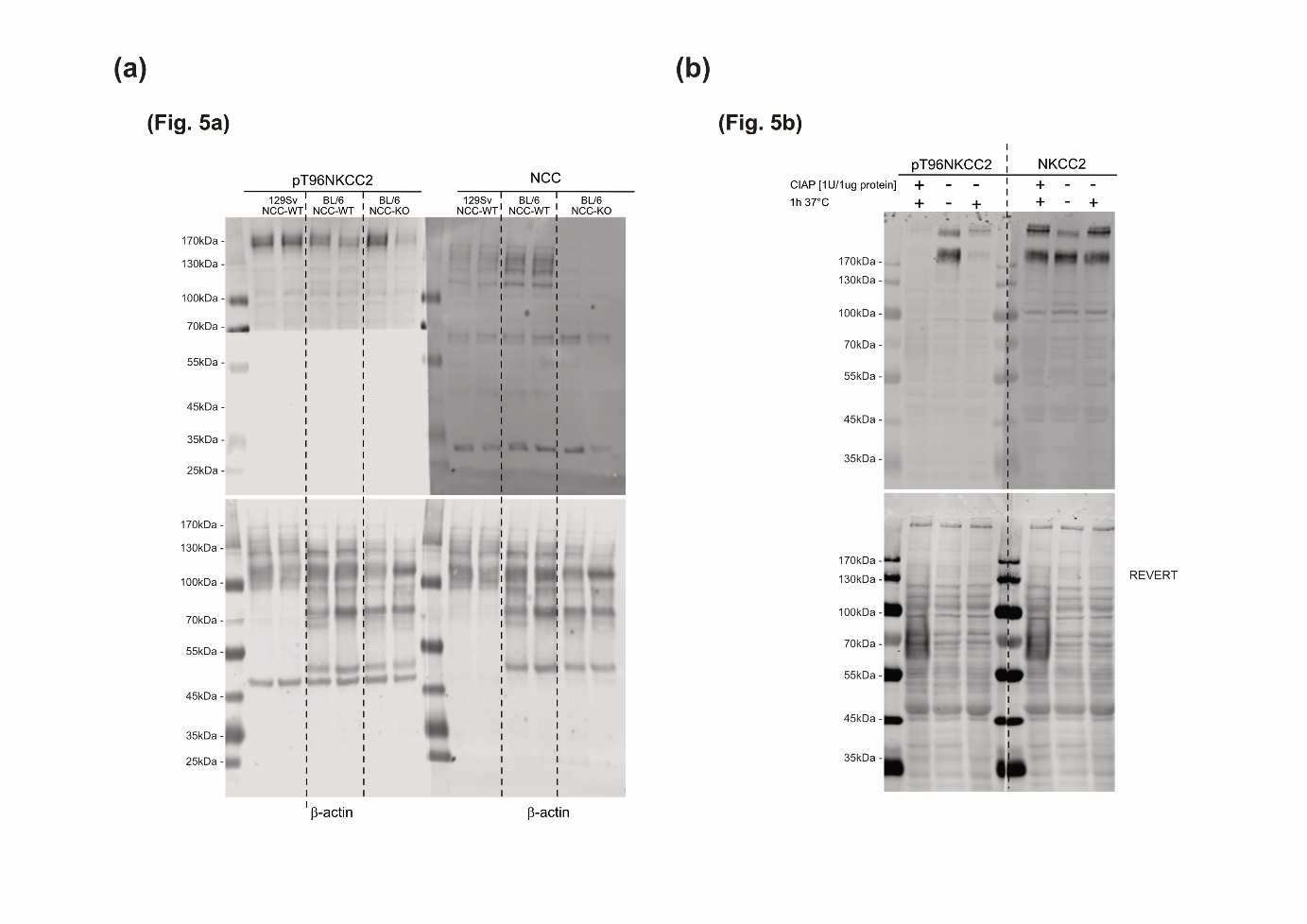** |
| --- |

**Supplementary Figure 13: Full gels of Western blots Fig. 5a and 5b**

**Supplementary Figure 14:**

| **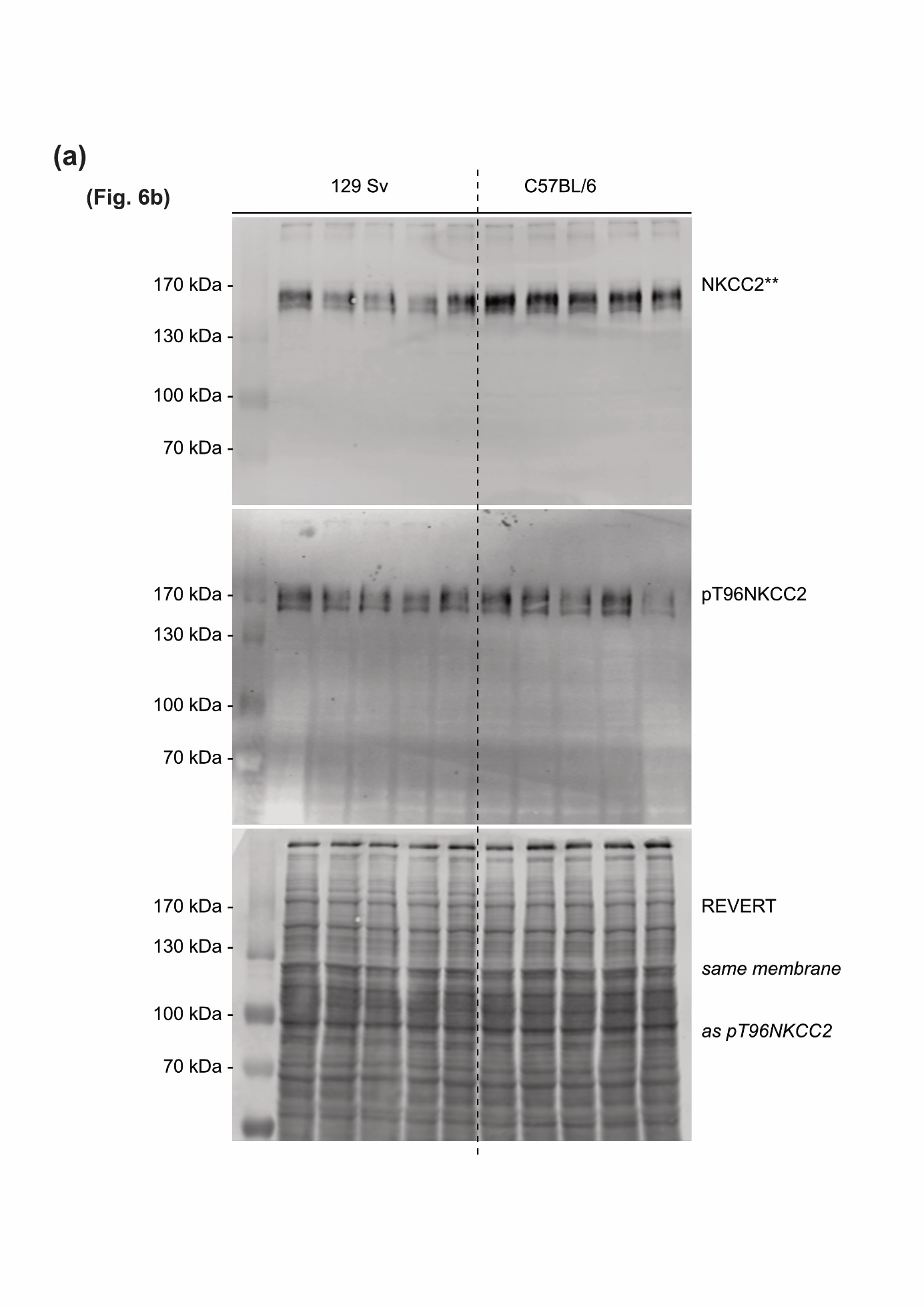** |
| --- |

**Supplementary Figure 14: Full gels of Western blots Fig. 6b**

**Supplementary Figure 15:**

| **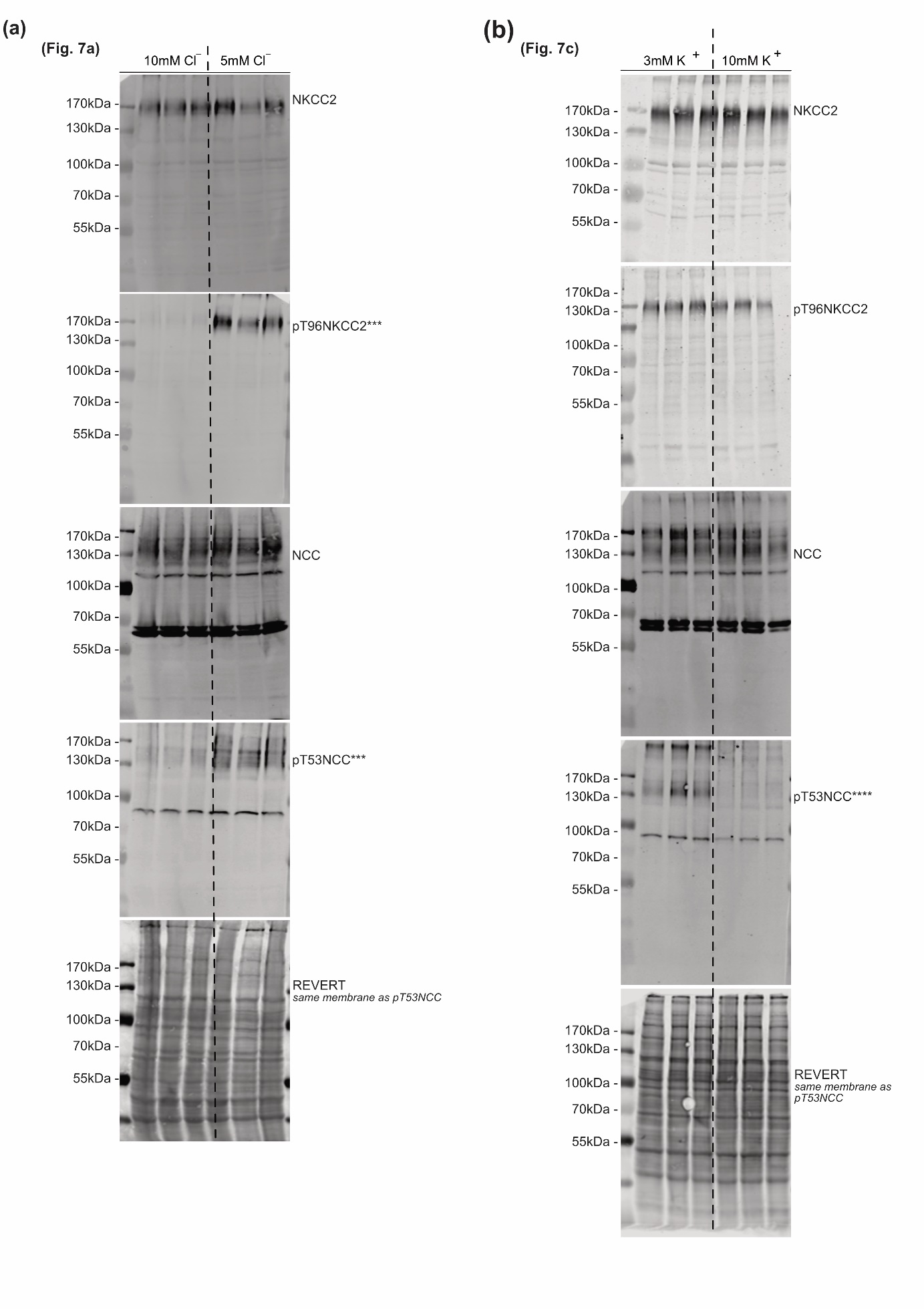** |
| --- |

**Supplementary Figure 15: Full gels of Western blots Fig. 7a and 7c**

**Supplementary Figure 16:**

| **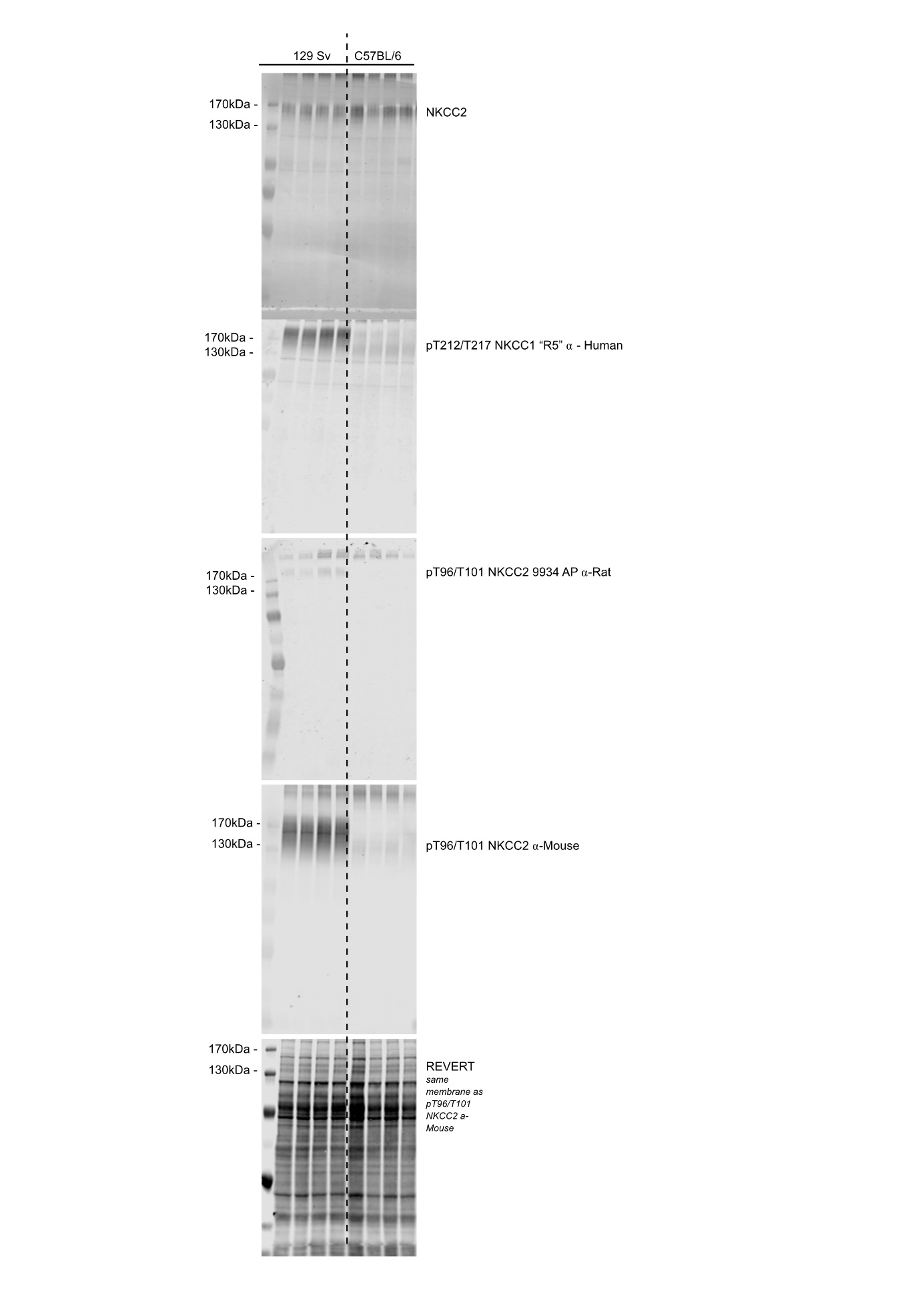** |
| --- |

**Supplementary Figure 16: Full gels of Western blots Supplementary Fig. 2**

**Supplementary Figure 17:**

| 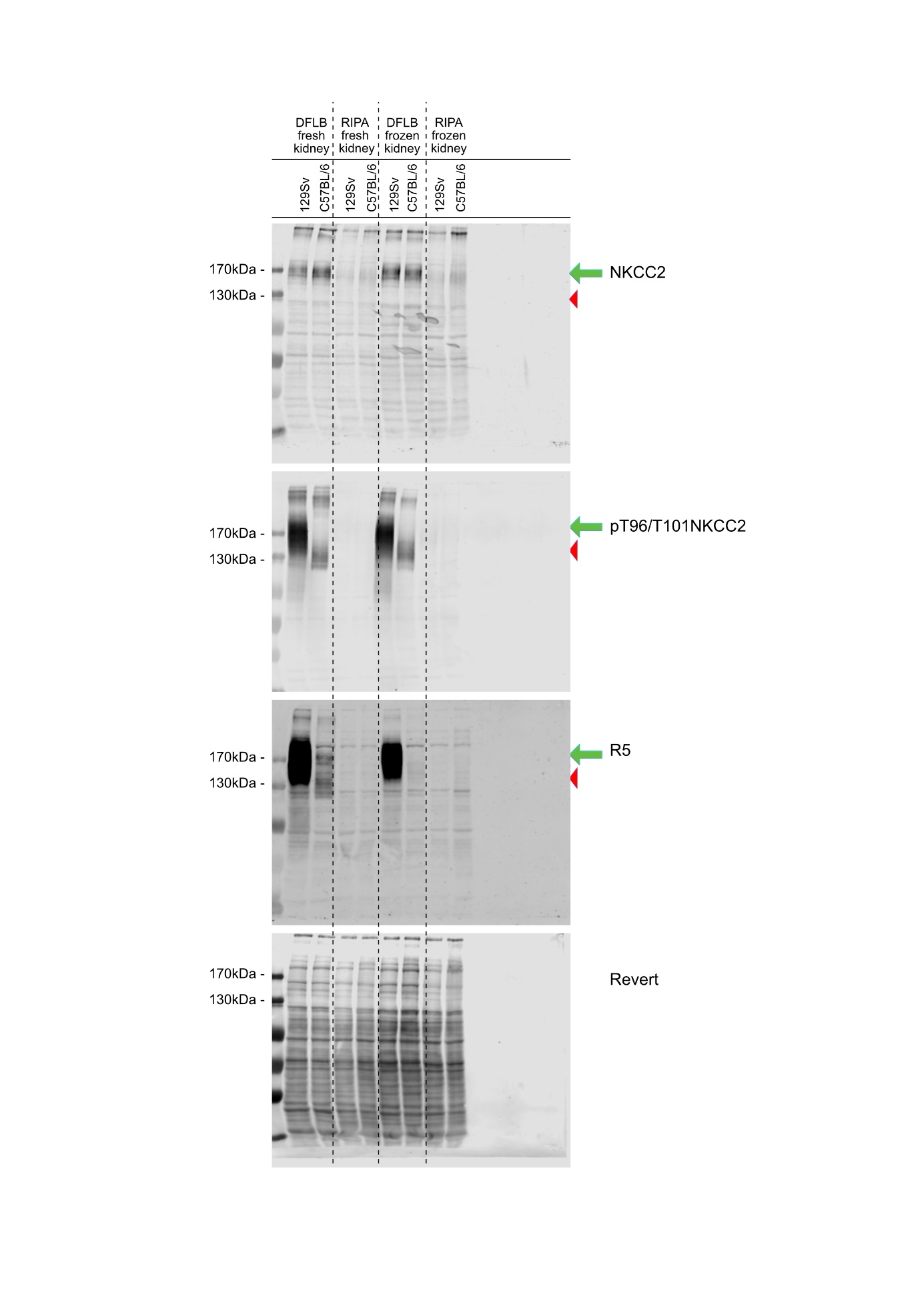 |
| --- |

**Supplementary Figure 17: Full gels of Western blots Supplementary Fig. 3**

**Supplementary Figure 18:**

| 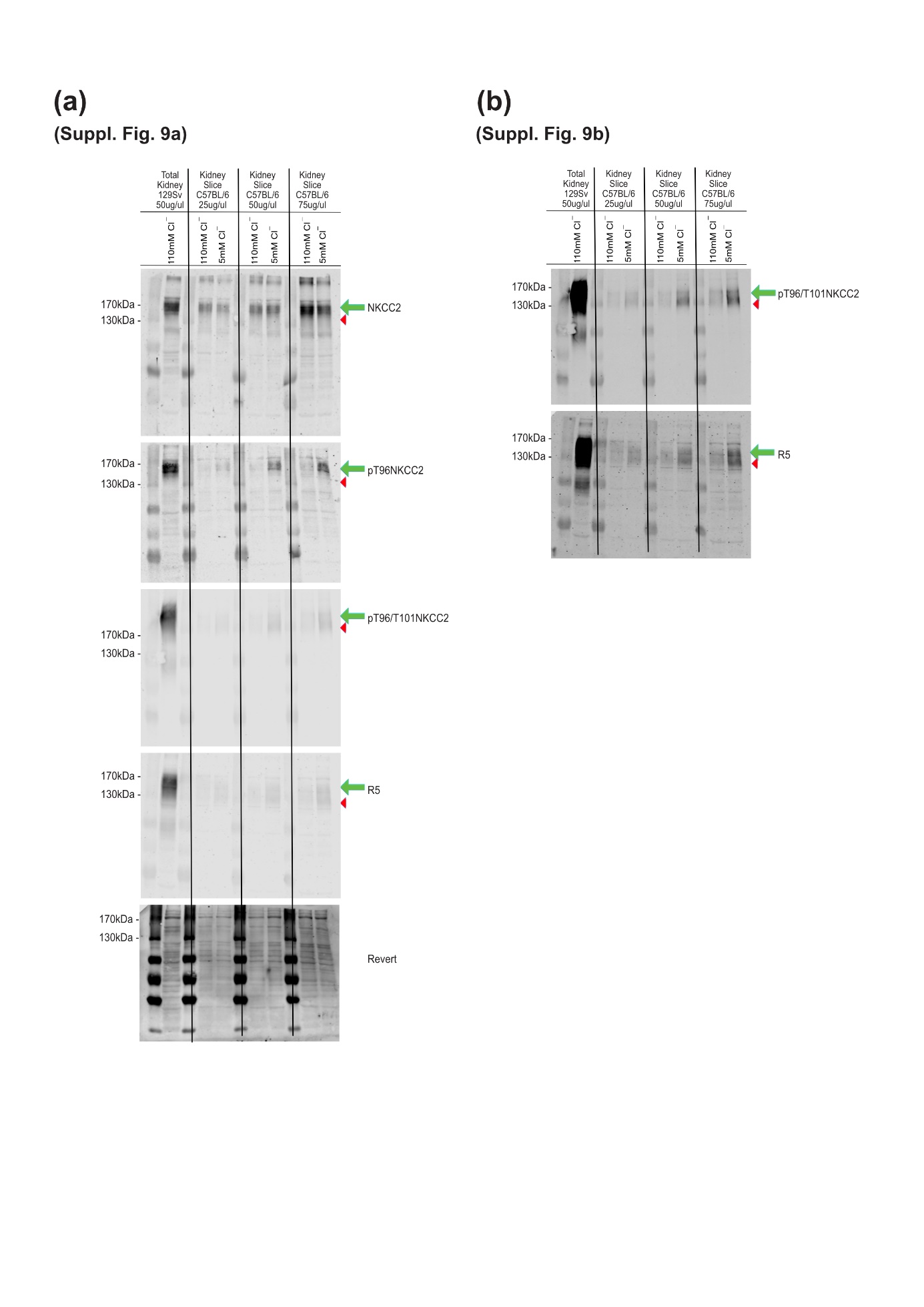 |
| --- |

**Supplementary Figure 18: Full gels of Western blots Supplementary Fig. 9**

**Supplementary References**

1 Daryadel A, Natale L, Seebeck P et al. Elevated FGF23 and disordered renal mineral handling with reduced bone mineralization in chronically erythropoietin over-expressing transgenic mice. *Sci Rep*. 2019;9(1). doi:10.1038/s41598-019-51577-z.

2 Rosenbaek LL, Kortenoeven MLA, Aroankins TS, Fenton RA. Phosphorylation decreases ubiquitylation of the thiazide-sensitive cotransporter NCC and subsequent clathrin-mediated endocytosis. *J Biol Chem*. 2014;289(19). doi:10.1074/jbc.M113.543710.

3 Rosenbaek LL, Rizzo F, MacAulay N, Staub O, Fenton RA. Functional assessment of sodium chloride cotransporter NCC mutants in polarized mammalian epithelial cells. *Am J Physiol - Ren Physiol*. 2017;313(2):F495–F504.

4 Rosenbaek LL, Rizzo F, Wu Q et al. The thiazide sensitive sodium chloride co-transporter NCC is modulated by site-specific ubiquitylation. *Sci Rep*. 2017;7(1). doi:10.1038/s41598-017-12819-0.

5 Rieg T, Tang T, Uchida S, Hammond HK, Fenton RA, Vallon V. Adenylyl cyclase 6 enhances NKCC2 expression and mediates vasopressin-induced phosphorylation of NKCC2 and NCC. *Am J Pathol*. 2013;182(1):96–106.

6 Sorensen M V., Grossmann S, Roesinger M et al. Rapid dephosphorylation of the renal sodium chloride cotransporter in response to oral potassium intake in mice. *Kidney Int*. 2013;83(5):811–824.

7 Wagner CA, Loffing-Cueni D, Yan Q et al. Mouse model of type II Bartter’s syndrome. II. Altered expression of renal sodium- and water-transporting proteins. *Am J Physiol - Ren Physiol*. 2008;294(6):1373–1380.

8 Penton D, Czogalla J, Wengi A et al. Extracellular K+ rapidly controls NaCl cotransporter phosphorylation in the native distal convoluted tubule by Cl−-dependent and independent mechanisms. *J Physiol*. 2016;594(21):6319–6331.

9 Flemmer AW, Giménez I, Dowd BFX, Darman RB, Forbush B. Activation of the Na-K-Cl cotransporter NKCC1 detected with a phospho-specific antibody. *J Biol Chem*. 2002;277(40):37551–37558.

10 Dimke H, Flyvbjerg A, Bourgeois S et al. Acute growth hormone administration induces antidiuretic and antinatriuretic effects and increases phosphorylation of NKCC2. *Am J Physiol - Ren Physiol*. 2007;292(2):F723–F735.

11 Penton D, Vohra T, Banki E et al. Collecting system–specific deletion of Kcnj10 predisposes for thiazide- and low-potassium diet–induced hypokalemia. *Kidney Int*. 2020;97(6):1208–1218.

12 Schnoz C, Carrel M, Loffing J. Loss of sodium chloride co-transporter impairs the outgrowth of the renal distal convoluted tubule during renal development. *Nephrol Dial Transplant*. 2020;35(3):422–432.

13 van der Hagen EAE, Lavrijsen M, van Zeeland F et al. Coordinated regulation of TRPV5-mediated Ca2+ transport in primary distal convolution cultures. *Pflugers Arch Eur J Physiol*. 2014;466(11):2077–2087.
